# Tailoring Oligomeric Plasticizers for Polylactide through Structural Control

**DOI:** 10.1021/acsomega.2c01160

**Published:** 2022-04-12

**Authors:** Wenxiang Xuan, Karin Odelius, Minna Hakkarainen

**Affiliations:** Department of Fibre and Polymer Technology, Kungliga Tekniska Högskolan, KTH, Teknikringen 58, SE 10044 Stockholm, Sweden

## Abstract

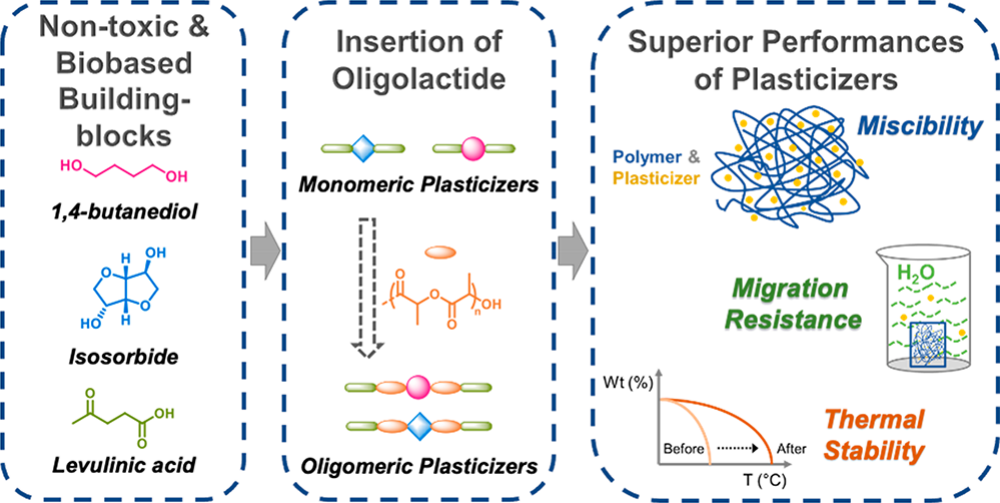

Structural variations (oligolactide segments, functionalized end groups, and different plasticizer cores) were utilized to tailor the performances of biobased plasticizers for polylactide (PLA). Six plasticizers were developed starting from 1,4-butanediol and isosorbide as cores: two monomeric (1,4-butanediol levulinate and isosorbide levulinate) and four oligomeric plasticizers with hydroxyl or levulinate ester end groups (1,4-butanediol-based oligolactide, isosorbide-based oligolactide, 1,4-butanediol-based oligomeric levulinate, and isosorbide-based oligomeric levulinate). Structural variations in plasticizer design were reflected in the thermal stability, plasticizing efficiency, and migration resistance. The monomeric plasticizer 1,4-butanediol levulinate decreased the glass-transition temperature of PLA from 59 to 16 °C and increased the strain at break substantially from 6 to 227% with 20 wt % addition. 1,4-Butanediol-based oligomeric levulinate exhibited better thermal stability and migration resistance, though the plasticizing efficiency was slightly lower (glass-transition temperature = 28 °C; strain at break = 202%). Compared to PLA films plasticized by plasticizers with flexible butanediol cores, those plasticized by plasticizers with rigid isosorbide cores exhibited higher Young’s modulus and thermal stability and lower plasticizing efficiency. Furthermore, plasticizers with levulinate ester end groups had improved thermal stability, plasticizing efficiency, and migration resistance compared to the corresponding plasticizers with hydroxyl end groups. Hence, a set of controlled structural variations in plasticizer design were successfully demonstrated as a potent route to tailor the plasticizer performances.

## Introduction

Plasticizers, as necessary additives for polymer processing and property adjustment, are widely employed at large volumes in the plastic industry.^1^ To move toward a more sustainable society, criteria such as designing and synthesizing new plasticizers utilizing green chemistry, by minimizing the dependence on fossil fuels, using and designing molecules with minimal hazards, and applying lifecycle thinking need to be addressed.^2^ Interesting examples of plasticizers include fishbone-like poly(methyl eleostearate),^3^ waste frying oil-based ethoxylated esters,^4^ diverse vegetable oil-based plasticizers,^5^ PLA oligomers,^6,7,19^ glyceryl lactate,^8^ and ketal-type esters^9^ that utilize biobased building blocks to ensure independence from fossil resources.^3−5^

For plasticizers, miscibility is an essential quality. Sufficient miscibility ensures constant properties of the plasticized material as the plasticizers remain homogeneously distributed over defined ranges of time, temperature, pressure, and composition.^10^ Phase separation,^11−13^ cold crystallization of the polymer,^14,15^ and crystallization of the plasticizers^16,17^ are identified as examples of potential consequences of inadequate miscibility. Several factors, including the molar mass, chemical structure, and molecular architecture, jointly determine the miscibility of the plasticizer in the polymer matrix. For instance, lactide and oligolactic acid (OLA) can both plasticize polylactide (PLA) efficiently because their high similarities with PLA in chemical and molecular structures lead to intrinsic and excellent miscibilities.^18−20^ However, variations in the structure can affect their performances, as illustrated by linear and cyclic OLA plasticizers that behave differently in PLA blends. Linear OLA plasticized PLA and accelerated the growth rate of spherulitics, while cyclic OLA also increased the nucleation rate and consequently the overall crystallization rate of PLA.^21^ Moreover, linear OLA resulted in larger water uptake and more rapid migration than cyclic OLA during hydrolytic degradation.^22^ Another studied structural variation is enantiomerism. Among d-lactide, l-lactide, and d,l-lactide, d,l-lactide demonstrated the strongest plasticizing efficiency and l-lactide had the lowest efficiency, whereas d-lactide exhibited the slowest migration rate.^23^ Moreover, the alcohol core in OLA is identified as an essential factor of structural variation. The utilization of ethanol, diethylene glycol, and trimethylolpropane cores to initiate the synthesis of OLAs influences the thermal stability, plasticization, and elastomeric behavior.^24^

Apart from the variations in the plasticizer structure, chemical functionalization of end groups in plasticizers is a flexible and common method to tune their properties. Hydroxyl groups are common end groups for plasticizers. For instance, both triethyl citrate and tributyl citrate are effective plasticizers for PLA, which after acetylation of the hydroxyl groups portrayed decreased volatility and rate of hydrolysis.^25^ Acetylation was also applied on hydroxyl-ended lactide oligomers, and improved thermal stability was realized.^24^ Another interesting plasticizer pair is hydroxyl end group polyethylene glycol (PEG) and its mono-ester of lauric acid with identical molar mass, where the hydroxyl end group PEG exhibited a higher plasticizing efficiency than PEG monolaurate.^20^ A carboxylic acid end group oligomer derived from diethyl bishydroxymethyl malonate was functionalized by triethylene glycol diamine to yield amides with increased compatibility with PLA due to hydrogen bonding.^26^ Furthermore, ketone end groups have interesting potential due to the diverse reactivity of ketone groups.^27^ The presence of ketones in the plasticizers has been shown to lead to reduced plasticizer volatility due to enhanced intermolecular interactions and increased miscibility with PLA as compared to a similar plasticizer without ketones.^28^ When the ketone is subsequently ketalized by ethylene glycol, the hydrophobicity and steric hindrance of the plasticizer increase, resulting in increased thermal stability and migration resistance of the plasticizer.^9^ Plasticizer design via functionalization can additionally be adjusted by utilizing various alkyl chain lengths in the plasticizer structure. For example, the plasticizing effect of maleate plasticizers increases, while the rate of biodegradation decreases with increasing number of carbon atoms in alkyl chains.^29^

The non-toxic, green platform, chemical levulinic acid (LeA) with ketone and carboxyl functionalities was anticipated to be a versatile base for plasticizers’ design. Here, it was utilized in combination with other biobased low toxicity building blocks, 1,4-butanediol and isosorbide.^30,31^ Moreover, LeA has demonstrated lower global warming potential than succinic acid, another top 12 value-added chemicals from biomass.^32,33^ Our hypothesis was that a set of controlled structural variations in plasticizer design (oligolactide segments, functionalized end groups, and plasticizer cores) would tailor performance characteristics, such as thermal stability and migration resistance, in combination with the plasticizing efficiency. At the same time, the introduction of oligolactide segments into the plasticizer structure could result in inherently miscible plasticizers and consequently strengthened the migration resistance.

## Experimental Section

### Materials

1,4-Butanediol (BTD, 99%, Riedel-de Haën), d-isosorbide (ISB, 98%, Sigma-Aldrich), levulinic acid (LeA, 97%, Sigma-Aldrich), l-lactide (98%, Sigma-Aldrich), *p*-toluene sulfonic acid monohydrate (PTSA·H_2_O, 98.5%, Sigma-Aldrich), and tin(II) 2-ethylhexanoate (Sn(Oct)_2_, 92.5%, Sigma-Aldrich) were utilized for the synthesis of plasticizer candidates. Ethyl acetate (EtOAc, analytical reagent grade, Fisher Scientific) and potassium carbonate (analytical reagent grade, Arcos) were employed in purifying and extracting the plasticizer candidates. Dichloromethane (HPLC grade, Fisher Scientific) and polylactide (5200D, Nature Works, *M*_n_ = 112,000 g/mol, *Đ* = 1.9) were used in solution casting to obtain neat and blended PLA films. Chloroform-*d* (99.8%, Cambridge Isotope Laboratories) and methanol (hypergrade for LC–MS, Merck) were used as solvents in nuclear magnetic resonance (NMR) and electrospray ionization mass spectrometry (ESI-MS) analyses, respectively. Water (hypergrade for LC–MS, Merck) was utilized in the hydrolytic aging experiments. Most chemicals were used as received, except BTD and Sn(Oct)_2_, which were preserved with the activated molecular sieves and l-lactide that was recrystallized in toluene twice before use.

### Synthesis of Monomeric and Oligomeric Plasticizer Candidates

The synthesis of the monomeric plasticizer candidate BTD-LeA was performed by mixing LeA (17.4 g, 0.15 mol), BTD (4.5 g, 0.05 mol), and PTSA·H_2_O (190 mg, 10^–3^ mol) under stirring in a 100 mL single-necked round-bottomed flask at 120 °C for 3 h at reduced pressure. A monomeric plasticizer candidate, ISB-LeA, was prepared and evaluated previously and included here for comparison.^28^ Two oligomeric plasticizer candidates with hydroxyl end groups were synthesized using ring-opening polymerization (ROP). In summary, for BTD-PLA-OH, BTD (1.8 g, 0.02 mol), l-lactide (10.1 g, 0.07 mol), and Sn(Oct)_2_ (28 mg, 7 × 10^–5^ mol) were mixed and allowed to react for 1.5 h and for ISB-PLA-OH, ISB (4.4 g, 0.03 mol), l-lactide (8.6 g, 0.06 mol), and Sn(Oct)_2_ (121 mg, 3 × 10^–4^ mol) were mixed and allowed to react for 2 h. Two additional oligomeric plasticizer candidates with levulinate end groups were synthesized in one-pot synthesis using two steps: first, ROP was performed as described above followed by end-group functionalization via Fischer esterification with LeA. In summary, for BTD-PLA-LeA: BTD (1.8 g, 0.02 mol), l-lactide (5.8 g, 0.04 mol), and Sn(Oct)_2_ (16 mg, 4 × 10^–5^ mol) were allowed to react for 1.5 h, followed by end-group functionalization with LeA (7.0 g, 0.06 mol) using PTSA·H_2_O (152 mg, 8 × 10^–4^ mol) as catalysts. For ISB-PLA-LeA: ISB (4.4 g, 0.03 mol), l-lactide (8.6 g, 0.06 mol), and Sn(Oct)_2_ (121 mg, 3 × 10^–4^ mol) were allowed to react for 2 h, followed by end-group functionalization with LeA (14 g, 0.12 mol) using PTSA·H_2_O (228 mg, 1.2 × 10^–3^ mol) as the catalyst. All ROP reactions were carried out in a 100 mL single-necked round-bottomed flask at 110 °C. For the subsequent Fischer esterification, upon cooling, LeA and PTSA·H_2_O were directly added into the batch and reacted at 120 °C at reduced pressure overnight. All the final reaction mixtures containing the synthesized plasticizer candidates were dissolved in EtOAc in a separation funnel, followed by the addition of K_2_CO_3_ (aq.). The pH of the separated water phase was adjusted to 9–10. The desired EtOAc phase was separated and further purified with water twice. Rotary evaporation at reduced pressure was used to remove EtOAc, and the synthesized plasticizer candidates were collected.

### Proton NMR Spectroscopy

The chemical structures of the synthesized plasticizer candidates were confirmed by proton NMR (^1^H NMR) spectroscopy. All analyses were conducted on a Bruker Avance 400 spectrometer at 25 °C with a frequency of 400 MHz. The peak from the chloroform residue in the solvent chloroform-*d* was taken as the internal reference. All data were processed using MestReNova v9.0.0 software.

### ESI-MS Analysis

All the synthesized plasticizer candidates were analyzed using a Finnigan LCQ ion trap mass spectrometer in the positive mode to examine their approximate molar mass. The plasticizer candidates were diluted in methanol and pumped in by a syringe with a speed of 20 μL/min. The ion source was set to 4.5 kV, and the capillary temperature was adjusted to 200 °C. The nebulizing gas was nitrogen.

### Preparation of PLA Films

The neat and blended PLA films with the plasticizer candidates were obtained by solution casting in Petri dishes with a diameter of 186 mm. In total, 4.0 g of neat PLA or PLA in combination with plasticizer candidates (20 or 30 wt % plasticizer candidates in total weight) were dissolved in 100 mL of dichloromethane. The polymer solution was stirred at 22 °C for at least 2 h and was then poured into Petri dishes. All Petri dishes were kept in a fume hood at 22 °C for at least 4 days, and the formed films were later removed from Petri dishes. The remaining solvent traces in the films were removed in a drying chamber at 60 °C under reduced pressure for 70 h. The blended PLA films were named according to the weight fraction of the plasticizer candidate in the film and the abbreviation of the plasticizer candidate name, for example, 20BTD-LeA.

### Differential Scanning Calorimetry

The thermal properties of the neat and blended PLA films and neat plasticizers were determined by a Mettler Toledo differential scanning calorimeter 820 module under a nitrogen atmosphere. The protocol for differential scanning calorimetry (DSC) included two scans. The first scan ran in sequences: 25 °C → 200 °C, hold for 2 min, 200 °C → −30 °C, hold for 2 min, followed by the second scan from −30 to 200 °C. Both heating and cooling rates were set as 10 °C/min and the midpoint of glass transition in the second scan was selected as the glass-transition temperature *T*_g_ for comparison. Triplicate samples were analyzed. The crystallinity was calculated by applying eq 1, where Δ*H*_m_, Δ*H*_cc_, Δ*H*_m_^0^, and *w*_PLA_ represent the enthalpy of melting, the enthalpy of cold crystallization, the enthalpy of melting for 100% crystalline PLA, and the weight percentage of PLA, respectively. The enthalpy of melting for 100% crystalline PLA used in calculations was 93.1 J/g.^34^
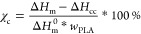
1

### Thermogravimetric Analysis

The thermal stabilities of the neat plasticizer candidates as well as the neat and blended PLA films were measured by a Mettler Toledo thermogravimetric analysis (TGA)/DSC 851e module instrument under a nitrogen atmosphere. The heating scan was set from 25 to 500 °C with a heating rate of 5 °C/min and a nitrogen flow rate of 50 mL/min. Triplicate samples were analyzed. The onset temperature corresponding to 5% weight loss (*T*_5_) was extracted and used for the comparisons.

### Tensile Testing

The mechanical performances of neat and blended PLA films were evaluated by tensile testing on an INSTRON 5944 module equipped with pneumatic grips. All the PLA-based films were cut into rectangular specimens with a constant width of 5 mm and a length of roughly 100 mm. The specimens were conditioned before testing for 40 h at RH 50 ± 5% and 23 ± 1 °C, as required in ASTM D618-13 (Standard Practice for Conditioning Plastics for Testing). A load cell of maximum 500 N was set at a crosshead speed of 20 mm/min, and the length between gauges was 20 mm. At least six specimens were measured for each film.

### Dynamic Mechanical Analysis

The homogeneity of plasticizer candidate distribution in all PLA films was assessed by dynamic mechanical analysis (DMA) (Q800, TA Instruments). Rectangular specimens (length: 10–15 mm; width: 5 mm; and thickness: 0.1–0.2 mm) were mounted in the tensile mode. All measurements were performed at 1 Hz frequency, and the temperature program scanning was from −50 to 120 °C with a heating rate of 3 K/min. The amplitude was 5 mm and the auto-strain was set to 125%. Duplicate samples were analyzed.

### Hydrolytic Aging Evaluation

Pieces of 1 cm × 1 cm of neat and blended PLA films were placed in a 20 mL sealed vial containing 10 mL of water and stored in a thermostatic oven at 60 °C for 1 day, 5 days, and 10 days, respectively. Triplicates were prepared at each time points. Air bubbles attached to the films after heating were driven away by manual vibration to ensure the intact contact of films with water.

### Mass Loss Measurement

The dry weights (*w*_1_) of neat and blended PLA films were measured after 1, 5, and 10 days of hydrolytic aging. All the PLA films were dried in a vacuum chamber at 60 °C for 3 days. Triplicate samples were analyzed. By comparing the initial weight of the films (*w*_0_), the mass loss (in percentage) was defined by eq 2
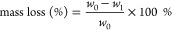
2

### Size Exclusion Chromatography

The molar mass of neat and blended PLA films before and after hydrolytic aging were analyzed by a GPCMAX (from Malvern) that has an auto-sampler, a PLgel 5 μm Guard column, and two PLgel 5 μm MIXED-D columns. The carrier solvent was chloroform containing 2 vol % toluene as an internal standard. The flow rate was set to 0.5 mL/min, and the temperature was set to 35 °C. A narrow polystyrene standard (*M*_n_ = 1200–400,000 g/mol) was used for calibration. Triplicate samples were analyzed.

## Results and Discussion

A series of plasticizer candidates with small structural variations were designed to evaluate the effects of the inclusion of oligolactide segments, in combination with variations in end groups (hydroxyl and LeA) and structural differences in the alcohol cores on the thermal, mechanical, and migration patterns of plasticized PLA. The plasticizer candidates were designed from potentially renewable chemicals using a flexible (1,4-butanediol) or rigid (isosorbide) core and combined with three different types of flanking groups, forming in total six plasticizers grouped into three different structural categories.

### Synthesis and Characterization of Plasticizer Candidates

**Figure 1:**
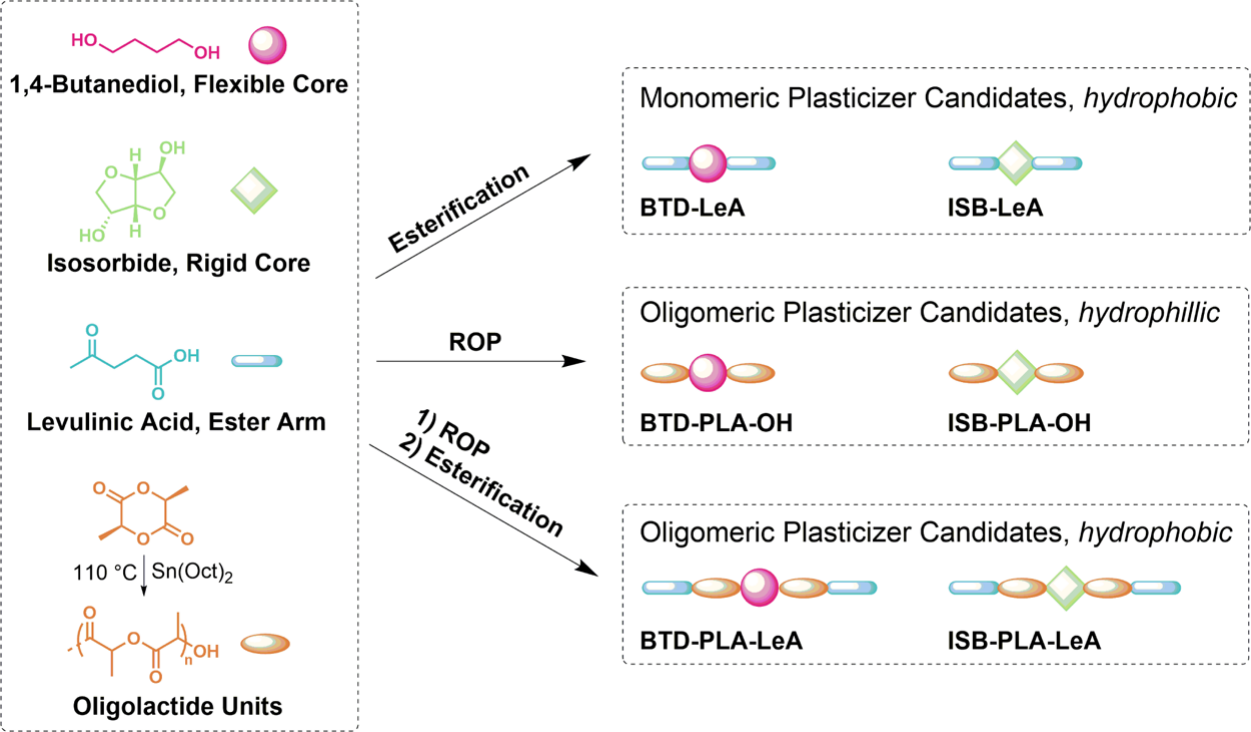
Scheme illustrating the plasticizer candidate structures.

The designed and synthesized plasticizer candidates for PLA are illustrated in Figure 1. BTD-LeA and ISB-LeA, two hydrophobic esters with structurally different alcohol cores were selected as monomeric plasticizer candidates. To tailor the properties of BTD-LeA and ISB-LeA, oligolactide segments were inserted into their structures by utilizing ROP of l-lactide with ISB or BTD as initiators followed by end-group functionalization via Fischer esterification using LeA. Consequently, two oligomeric plasticizer candidates with oligolactide segments and levulinate end groups, ISB-PLA-LeA and BTD-PLA-LeA, were obtained. To investigate the effects of levulinate end groups on the properties of oligomeric plasticizer candidates, another two oligomeric plasticizer candidates with hydroxyl end groups, BTD-PLA-OH and ISB-PLA-OH, were prepared by ROP of l-lactide from BTD or ISB, respectively, without LeA end-group functionalization. The ratios of l-lactide to initiator in ROP reactions were adjusted to target a similar number-averaged molar mass (*M*_n_) for the four oligomeric plasticizer candidates, regardless of the end-group type. All the obtained plasticizer candidates were liquids, except that BTD-LeA was a pale-yellow solid.

#### Chemical Structure of Plasticizer Candidates

**Figure 2:**
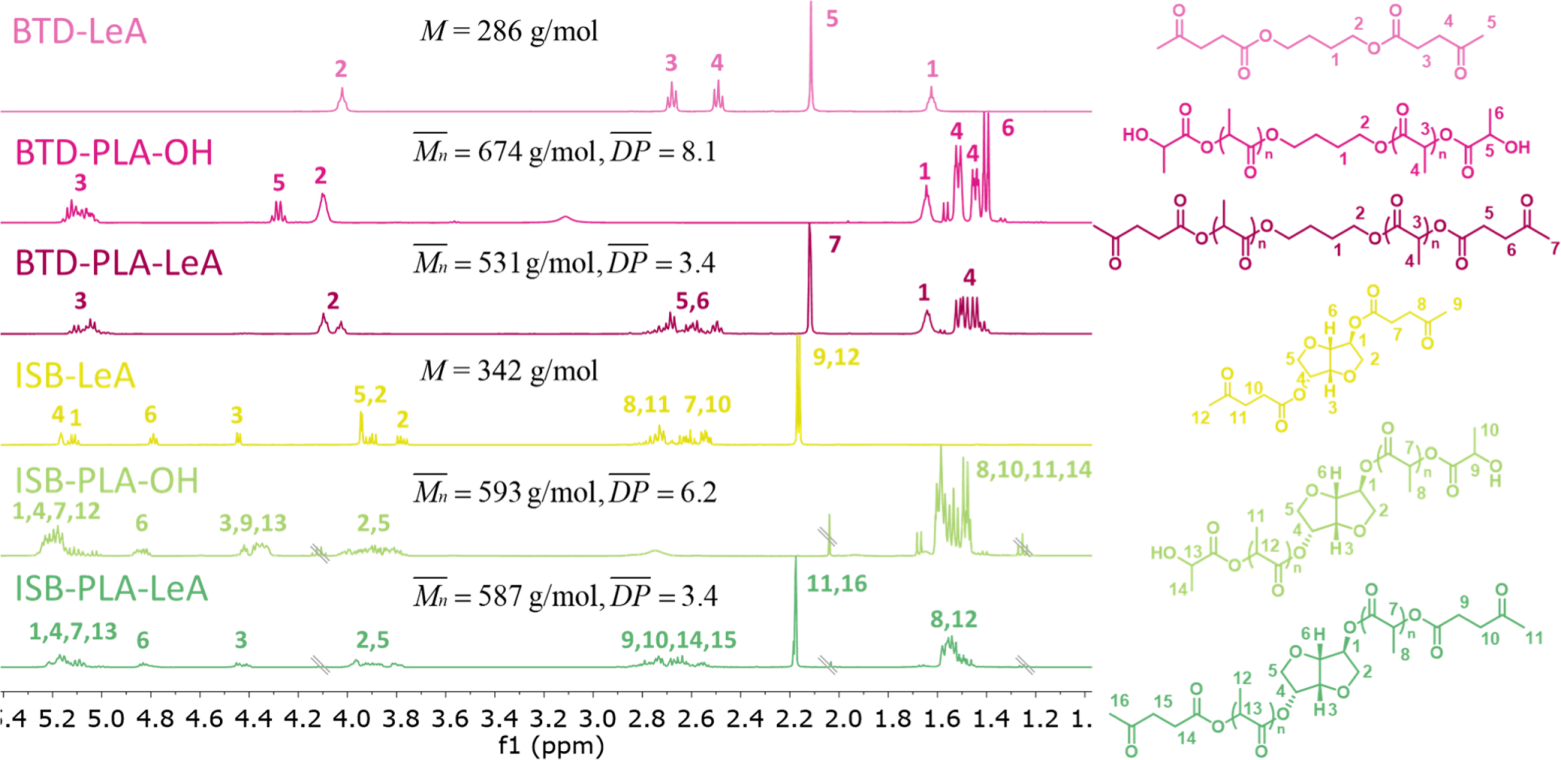
^1^H NMR spectra of BTD-LeA, BTD-PLA-OH, BTD-PLA-LeA, ISB-PLA-OH, and ISB-PLA-LeA.

**Figure 3:**
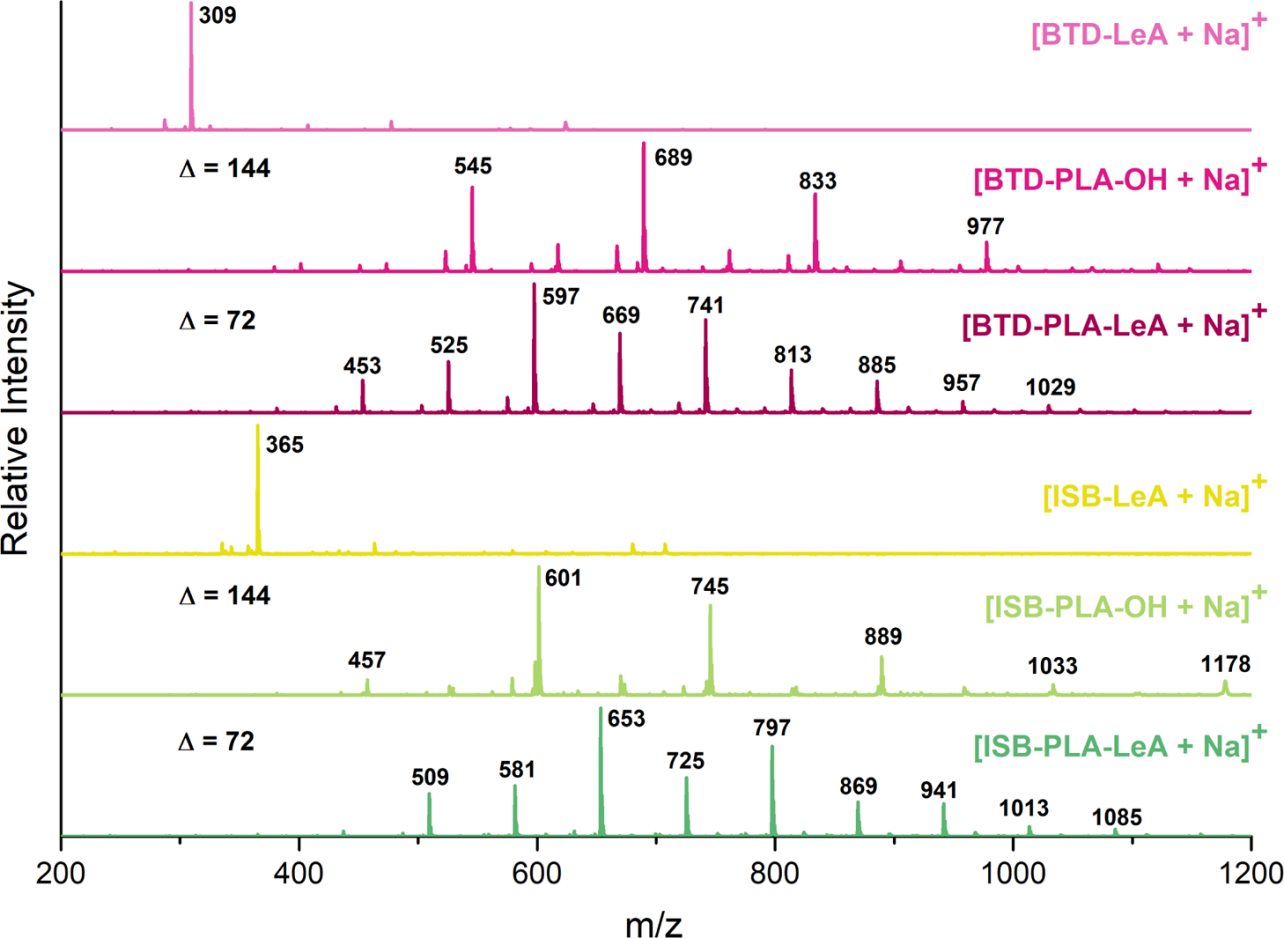
ESI-MS spectra of BTD-LeA, BTD-PLA-OH, BTD-PLA-LeA, ISB-LeA, ISB-PLA-OH, and ISB-PLA-LeA.

^1^H NMR and ESI-MS were utilized to determine the chemical structure of the synthesized plasticizer candidates, except for BTD-LeA, which has previously been characterized.^28^ The chemical shifts and related proton assignments are summarized in Figure 2, and individual spectra are found in Figures S1–S5. The ^1^H NMR results suggested that both hydroxyl groups of the alcohol cores BTD and ISB were either functionalized with LeA or had initiated the ROP of l-lactide. The presence of levulinate end groups in the oligomeric plasticizer candidates BTD-PLA-LeA and ISB-PLA-LeA and the hydroxyl end groups in BTD-PLA-OH and ISB-PLA-OH was confirmed by ^1^H NMR. The *M*_n_ of the four oligomeric plasticizer candidates were calculated and are displayed in Figure 2. The molar mass and chemical structure information of the plasticizer candidates were further supported by ESI-MS (Figure 3). The monosodium adducts of the synthesized plasticizer candidate were detected, and the ESI-MS spectra indicated that the four oligomeric plasticizer candidates were in the same molar mass range.

#### Thermal Stability of Plasticizer Candidates

**Figure 4:**
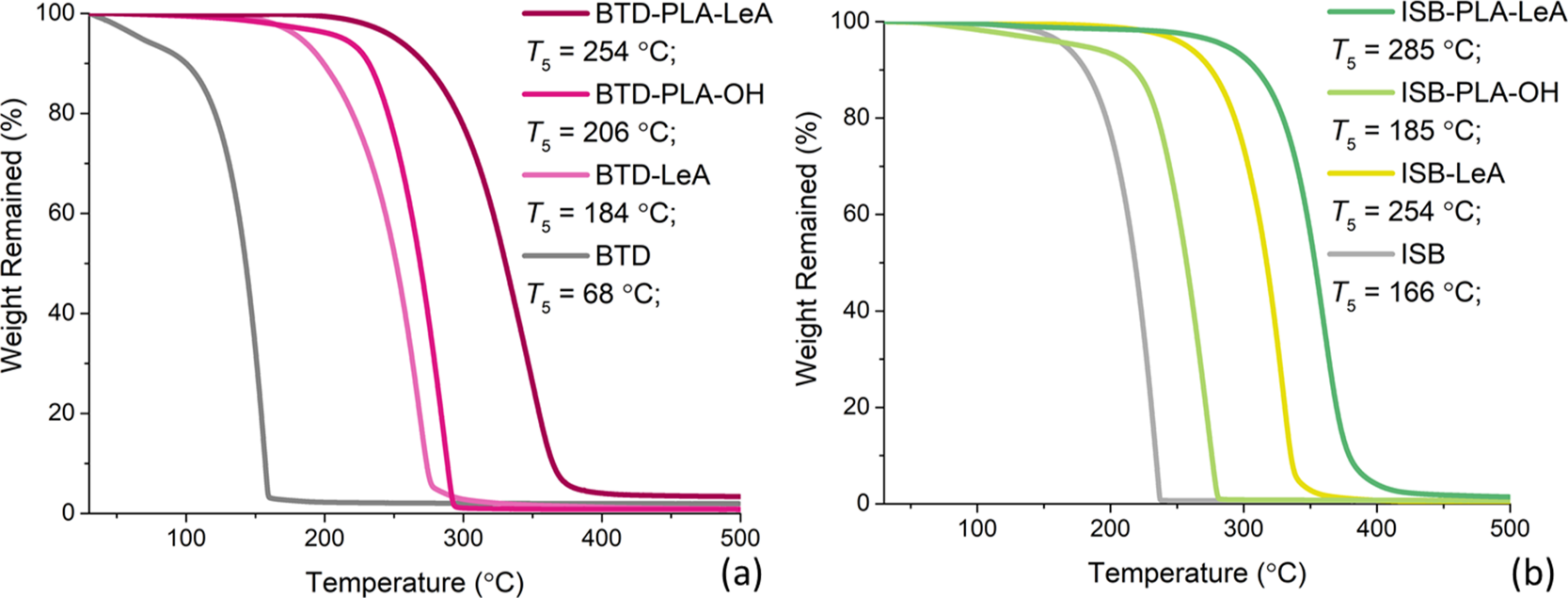
TGA traces of neat BTD and BTD-based plasticizer candidates (a) and TGA traces of neat ISB and ISB-based plasticizer candidates (b).

To function as a plasticizer, the plasticizer candidates should, among other characteristics, be thermally stable during the processing of PLA and exhibit low volatility. Hence, TGA was used to examine the volatility and thermal stability of the plasticizer candidates. The TGA traces are plotted in Figure 4, and the temperature, where 5 wt % mass loss occurred (*T*_5_), was taken as an indicator to assess and compare the thermal stabilities of the plasticizer candidates. The plasticizers need to be thermally stable at or slightly above the melting temperature of polymers. As seen in Figure 4, starting materials BTD and ISB were characterized by TGA as well, and all plasticizer candidates demonstrated sufficient thermal stabilities as their *T*_5_ values were higher than the melting temperature of the used PLA (151 °C). The six plasticizer candidates had structural variations that significantly affected the thermal stability of the plasticizer candidates. First of all, the inserted oligolactide segments increased the thermal stabilities of the plasticizer candidates. Compared to the neat alcohol cores BTD and ISB, oligomeric plasticizer candidates BTD-PLA-OH and ISB-PLA-OH with oligolactide units exhibited higher *T*_5_ values (*T*_5_ increased by 138 and 19 °C, respectively), and their TGA traces were shifted toward the higher temperature region. The same trend was also observed for the plasticizer candidates BTD-LeA with BTD-PLA-LeA or ISB-LeA with ISB-PLA-LeA with levulinate end groups. An increase of 70 °C or 31 °C in the *T*_5_ value was defined for BTD-PLA-LeA and ISB-PLA-LeA, respectively, as an effect of oligolactide insertion. The increase in thermal stability when oligolactide segments were introduced was likely due to the increased molar mass and possibly higher thermal stability of the oligolactide segments because PLA had a *T*_5_ value of 316 °C. Moreover, end-group functionalization with LeA was another critical factor contributing to the enhancement of the thermal stability of the plasticizer candidates. A large difference in thermal stability caused by the different end -groups (hydroxyl end groups vs LeA-functionalized ester end groups) was considered to be caused by the active hydroxyl groups enabling, for example, transesterification reactions. Therefore, the *T*_5_ values of the oligomeric plasticizer candidates BTD-PLA-OH and ISB-PLA-OH were lower than their LeA-functionalized oligomeric plasticizer candidates BTD-PLA-LeA and ISB-PLA-LeA. A similar decreased thermal stability because of a higher amount of active groups was previously observed in eugenol-based oligomeric plasticizers, as the *T*_5_ value of plasticizers with a higher phenolic content was 20 °C lower than that of the plasticizer with less phenolic content.^35^ Ketone groups were previously proven to increase the thermal stability of plasticizers.^28^ In general, the thermal stabilities of the synthesized plasticizer candidates were quite comparable to other PLA plasticizers. For instance, plasticizers, ethylene glycol dilevulinate (ED) and glycerol trilevulinate (GT), just like BTD-LeA, were made of saturated alkyl alcohols, but their *T*_5_ values were lower than that of BTD-LeA (184 °C for BTD-LeA, 140 °C for ED and 164 °C for GT).^9^ Similar to the oligolactide structures here, lactic acid oligomers (*M*_n_ = 671–957 g/mol) were utilized to plasticize PLA, and their *T*_5_ values were located between 179 and 214 °C.^6^

### Thermal Properties of PLA Films Containing Plasticizer Candidates

**Figure 5:**
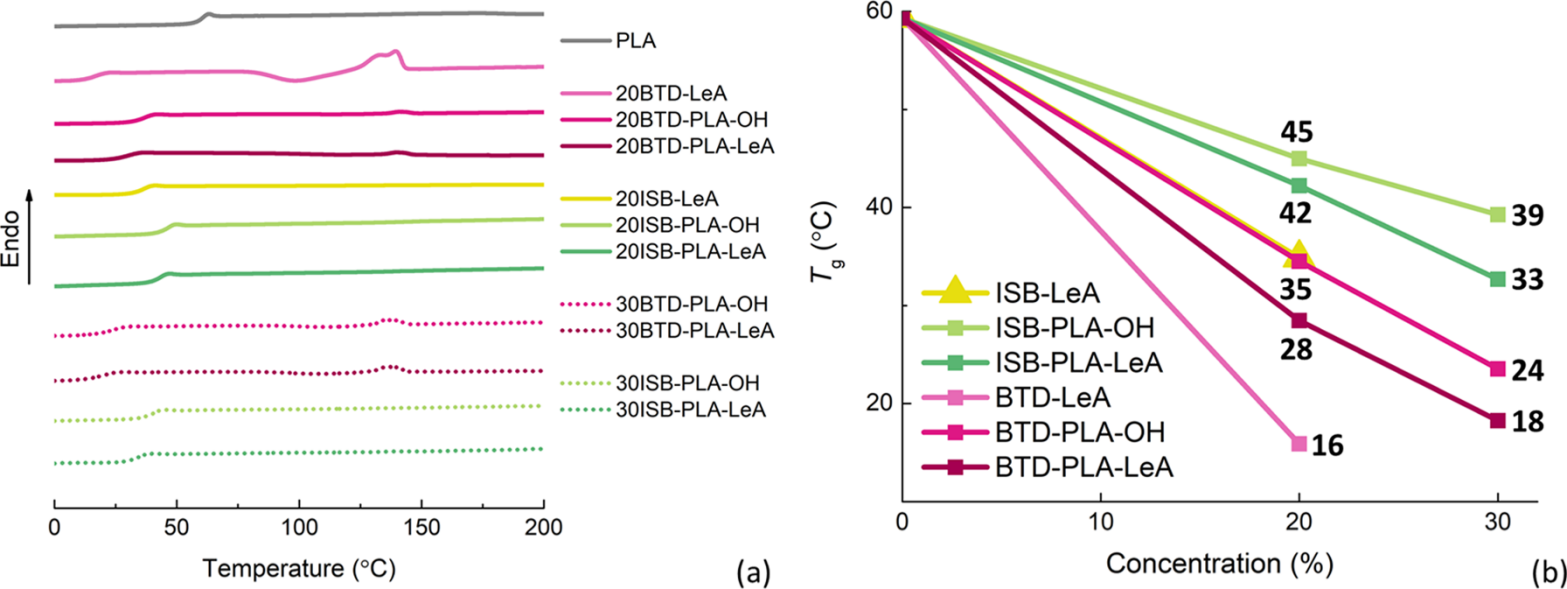
DSC traces of neat PLA and PLA films with the plasticizer candidates (a) and a plot of *T*_g_ as a function of plasticizer candidate concentration (b).

According to the IUPAC definition of a plasticizer, a plasticizer is a substance or material incorporated in a material (usually plastic or elastomer) that increases its flexibility, workability, or distensibility.^36^ By this definition, a plasticizer may increase the strain at break, lower the *T*_g_, or decrease the Young’s modulus of the material. Furthermore, successful plasticization is usually realized by miscible plasticizers. Therefore, the neat plasticizer candidates and PLA films containing 20 wt % of the monomeric plasticizer candidates or 20 or 30 wt % of the oligomeric plasticizer candidates were characterized by DSC to evaluate their thermal properties and plasticization efficiencies (Table S1). As described in Figure 5a, decreased *T*_g_ was seen in all the DSC thermograms, and *T*_g_ continued to decrease with increasing concentration of the oligomeric plasticizer candidates (Figure 5b), indicating that all six plasticizer candidates were at least partially miscible and served as plasticizers for PLA. The experimental miscibility was further compared to the theoretical values by applying the Fox equation (Table S1). Although the *T*_g_ values obtained when applying the Fox equation were slightly higher than those obtained from DSC, the declining trends and degree of *T*_g_ decrease obtained theoretically and experimentally were comparable. The small deviation between the theoretical and experimental *T*_g_ values is likely explained by slightly lower secondary interactions in the blends compared with those theoretically predicted.^37^ The structural variations existing in the six plasticizers led to different plasticizing efficiencies. For example, the inserted oligolactide segments increased the *M* of the plasticizer and subsequently slightly inhibited the plasticizing efficiency. At a concentration of 20 wt %, the monomeric plasticizer BTD-LeA reduces the *T*_g_ of PLA from 59 to 16 °C, which is a larger reduction in *T*_*g*_ compared to the oligomeric plasticizer BTD-PLA-LeA, which lowered the *T*_g_ from 59 to 28 °C. In contrast to the influence of the inserted oligolactide segments, the end-group functionalization with LeA enhanced the plasticizing efficiency of the plasticizers. For instance, the *T*_g_ of PLA was further decreased by 7 °C when comparing 20 wt % BTD-PLA-LeA with 20 wt % BTD-PLA-OH. The four oligomeric plasticizers had similar *M*_n_, but likely the more polar hydroxyl end groups lead to a significantly higher *T*_*g*_ for the oligomeric plasticizers with hydroxyl end groups compared with the plasticizers that were end-group-functionalized with LeA. This was subsequently reflected in the *T*_*g*_ of the prepared blends. The same trend was found in the plasticizer pair ISB-PLA-OH and ISB-PLA-LeA as well, yet with consistently higher *T*_g_ values due to the rigid ISB core. The flexible and linear BTD core, thus, demonstrated stronger capability to lower the *T*_g_ as compared to the rigid and cyclic ISB core. In addition, all the films had comparable initial crystallinity within the range from 21 to 26% for the plasticized blends and 31% for neat PLA (Table S1).The slightly higher degree of crystallinity for neat PLA is explained by the higher PLA content when no plasticizer was added. During the second heating scan, only films plasticized by BTD-PLA-OH and BTD-PLA-LeA exhibited melting peaks with very low crystallinities below 2%. Specifically, the film plasticized by 20 wt % monomeric plasticizer BTD-LeA demonstrated a cold crystallization-induced melting procedure at 140 °C. Compared to the previously reported PLA plasticizers, the plasticizing efficiency of the six synthesized plasticizers were acceptable. For instance, with the addition of 15 wt % oligomeric plasticizer lactic acid oligomers (*M*_n_ = 671–957 g/mol), the *T*_g_ of PLA decreased from 59 to 36–39 °C.^6^ At 20 wt % plasticizer concentration, one of the above lactic acid oligomers (*M*_n_ = 957 g/mol) just lowered the *T*_g_ slightly more down to 34 °C.^7^ The oligomeric plasticizer polyethylene glycol (PEG) can efficiently plasticize PLA as well. With the addition of 20 wt % PEG (*M*_n_ = 400–1500 g/mol), the *T*_g_ of PLA was decreased to 12–38 °C.^38,39^ A stronger plasticizing efficiency was demonstrated by 20 wt % monomeric plasticizer ED and GT in which the *T*_g_ of PLA was lowered to 15 °C or 26 °C, respectively.^9^

### Thermal Stability of Plasticized PLA Films

**Figure 6:**
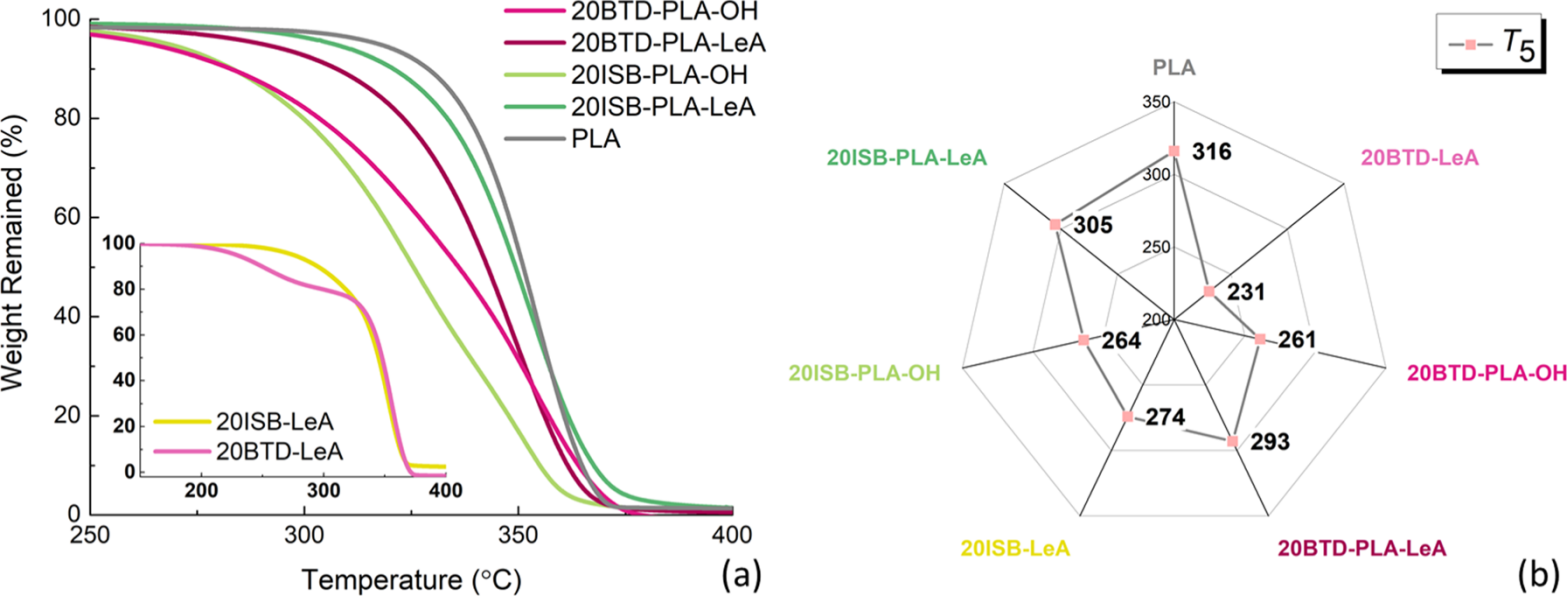
TGA traces (a) and a radar map of *T*_5_ values (b) of neat PLA and plasticized PLA films.

The neat PLA and plasticized PLA films containing 20 wt % of plasticizers were evaluated by TGA to determine their thermal stability (Figure 6a), and the *T*_5_ values were extracted for comparison and are mapped in Figure 6b. The same trends in thermal stability of the plasticized PLA films as for the neat plasticizers could be found. The films plasticized by LeA-functionalized oligomeric plasticizers (BTD-PLA-LeA and ISB-PLA-LeA) had higher thermal stability with higher *T*_5_ values than the films plasticized by oligomeric plasticizers with hydroxyl end groups (BTD-PLA-OH and ISB-PLA-OH). It is known that hydroxyl groups can promote the thermal degradation of PLA and therefore the thermal stability of PLA can be improved by blocking the hydroxyl end group of chains.^40,41^ The functionalization of the end groups of the plasticizer with LeA is thus likely beneficial for the stability of plasticized PLA during processing. Moreover, by comparing the alcohol cores, the plasticizers based on ISB had higher thermal stabilities than those derived from BTD. This was probably caused by the lower volatility and higher thermal stability of ISB and ISB-LeA compared to BTD and BTD-LeA. Unlike the TGA trace of the monomeric plasticizer BTD-LeA, where the degradation consisted of two individual degradation steps, first of BTD-LeA, followed by PLA, the PLA blends with oligomeric plasticizers degraded within a wide range of temperatures, and no clear steps of degradation could be seen. Compared to the PLA film plasticized by 20 wt % lactic acid oligomers (*M*_n_ = 957 g/mol, *T*_5_ = 265 °C),^7^ the PLA films plasticized by the four oligomeric plasticizers exhibited similar or higher *T*_5_ values.

### Mechanical Properties of Plasticized PLA Films

**Figure 7:**
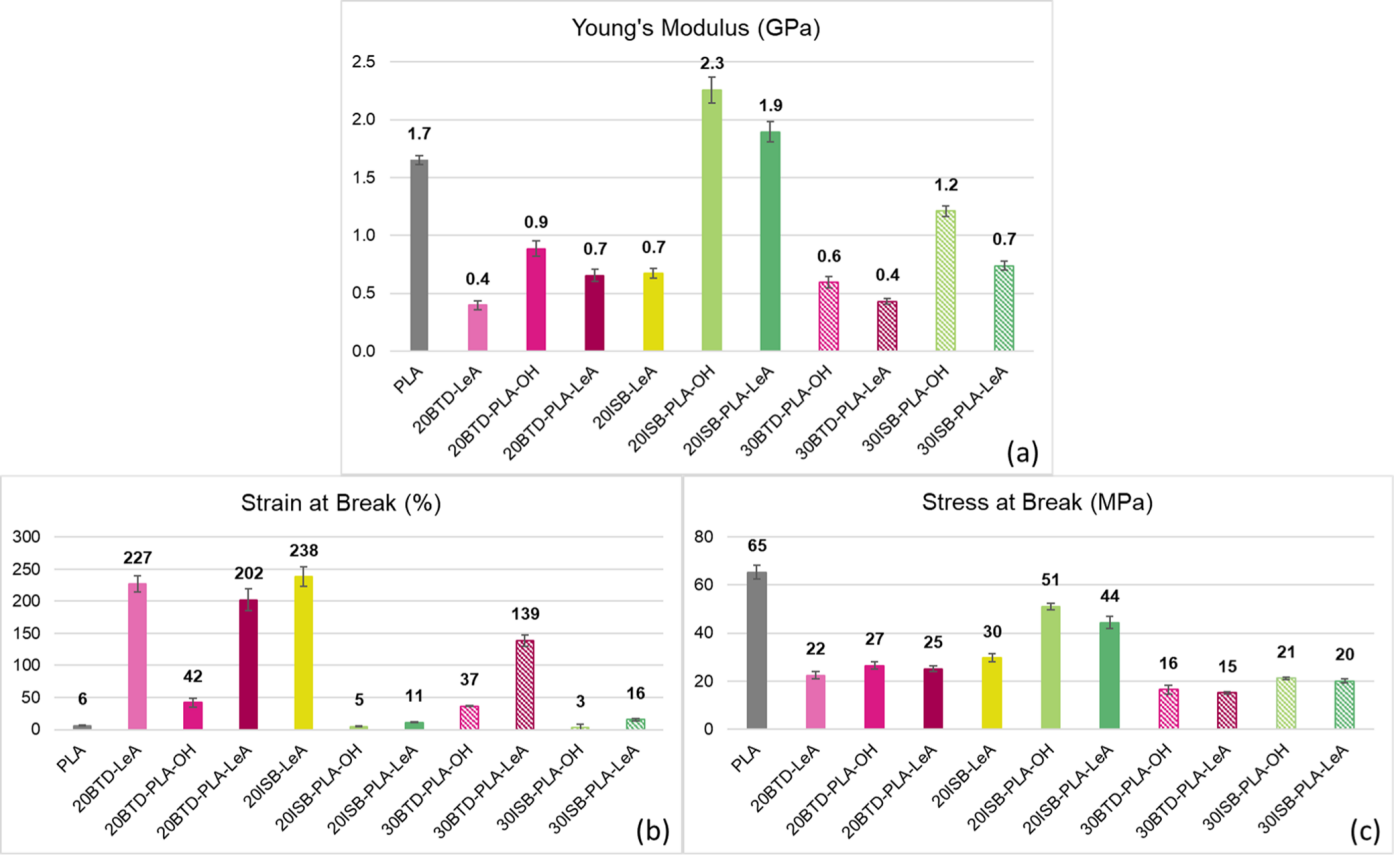
Mechanical properties of neat PLA and plasticized PLA films with plasticizer candidates: Young’s modulus (a), strain at break (b), and stress at break (c).

In general, the plasticizers provide more free volume to the plasticized system, which leads to an increase in chain mobility, reflected by the reduced Young’s modulus and stress at break and more importantly, the increased strain at break. Hence, the mechanical properties of neat PLA and plasticized PLA films were evaluated by tensile testing (Figures 7, S6–S16). The effects of structural variation in plasticizers, including the insertion of oligolactide segments, the functionalization of hydroxyl end groups with LeA, and the selection of the alcohol core on the mechanical properties of the plasticized PLA films were investigated. The mechanical properties of the plasticized PLA films with the LeA end groups with or without oligolactide segments varied notably. After that the insertion of oligolactide segments, Young’s modulus and stress at break increased 1.2 GPa and 14 MPa, respectively, for 20ISB-PLA-LeA as compared to 20ISB-LeA (Figure 7a,c). However, the insertion of oligolactide segments resulted in an increase of the molar mass of the plasticizer; thus, the plasticizing efficiency decreased, as reflected by the lower strain at break (238% vs 11% for 20ISB-LeA vs 20ISB-PLA-LeA) (Figure 7b). The *T*_g_ of the plasticized films supported the decrease in the plasticizing efficiency of the plasticizers because of the insertion of oligolactide segments. The second structural variation, the functionalization of the hydroxyl end groups with LeA altered the performances of the plasticizers as well, where the Young’s modulus decreased and strain at break increased. Compared to 20BTD-PLA-OH, the Young’s modulus of 20BTD-PLA-LeA dropped from 0.9 to 0.7 GPa, while the strain at break increased from 42 to 202%, suggesting a higher degree of plasticization after functionalization with LeA. A larger decrease of *T*_g_ values was observed in PLA films plasticized by LeA-functionalized oligomeric plasticizers, supporting the inferior plasticizing efficiency of the oligomeric plasticizers with hydroxyl end groups. The third structural variation was the rigidity of the alcohol core of the plasticizers, which significantly affected the mechanical properties of the plasticized PLA films. The monomeric plasticizer BTD-LeA demonstrated typical plasticizer behavior, increasing the strain at break from 6 to 227%, reducing the Young’s modulus from 1.7 to 0.4 GPa, and lowering the stress at break value from 65 to 22 MPa with 20 wt % addition. Compared to the flexible and linear alcohol core of BTD-LeA, ISB-LeA had a rigid and cyclic core, which resulted in a slightly higher Young’s modulus (0.7 GPa) and stress at break (30 MPa) at 20 wt %. It has previously been reported that increased Young’s moduli were observed for polymers due to the presence of ISB units.^42,43^ Although the *T*_g_ of 20ISB-LeA was 19 °C higher than that of 20BTD-LeA, the strain at break of 20ISB-LeA was close to that of 20BTD-LeA (238% vs 227%). This could have been caused by the solid state of BTD-LeA restricting its performance in increasing the strain at break. In addition, when increasing the concentration of the four oligomeric plasticizers from 20 to 30 wt %, a further increase in the strain at break of plasticized films was quite low or the strain at break decreased, indicating miscibility limitation in the excessive presences of oligomeric plasticizers, that is, phase separation likely occurred. Compared to other PLA plasticizers, the performances of the plasticizers BTD-LeA and BTD-PLA-LeA were encouraging. Monomeric plasticizers, EG and GT, increased the strain at break of PLA from 5 to 546 and 470% with 20 wt % addition.^9^ These values were realized for amorphous plasticized blends, compared to almost 30% crystallinity existing in the films plasticized by 20 wt % BTD-LeA and BTD-PLA-LeA. Moreover, strain at break values of 71 and 235% were achieved by 20 wt % PEG 400 and PEG 1500, respectively. Young’s moduli were 0.5–0.6 GPa and values of strain at break were between 16 and 22 MPa.^38^ Additionally, 20 wt % lactic acid oligomers (*M*_n_ = 957 g/mol) increased the strain at break value of PLA from 4 to 301%.^7^

### Dynamic Mechanical Analysis

**Figure 8:**
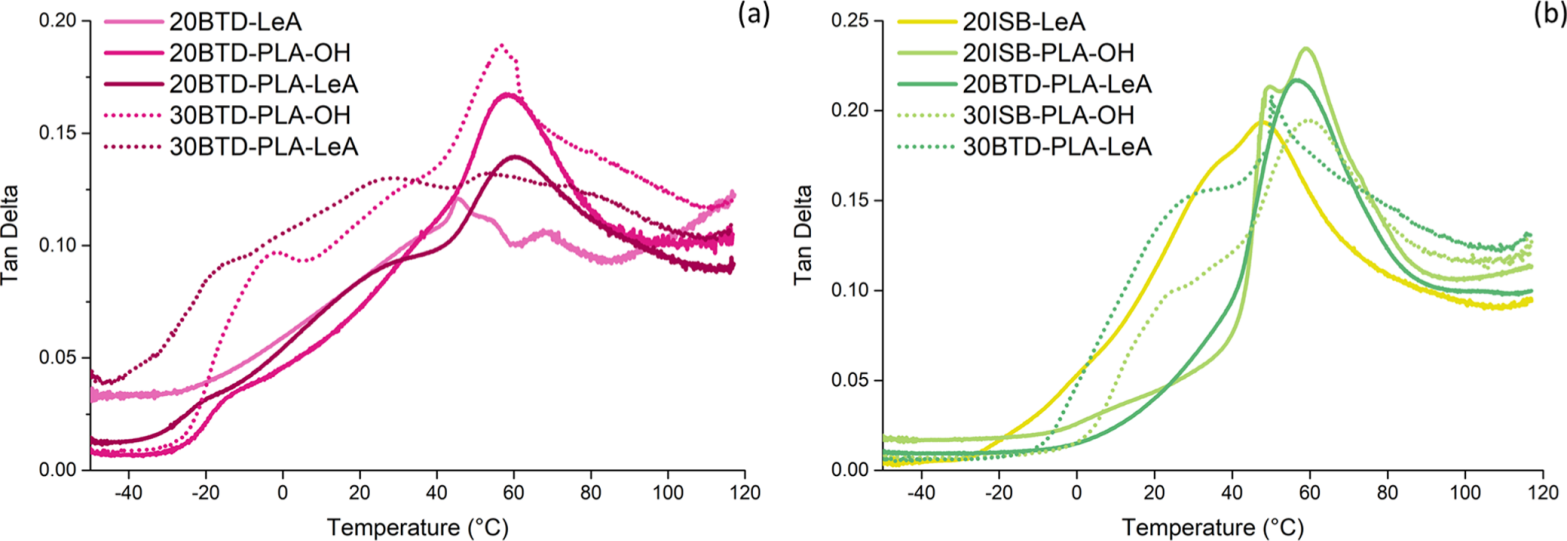
Temperature dependence of tan δ for plasticized PLA films measured at 1 Hz: BTD-based plasticizers (a) and ISB-based plasticizers (b).

The homogeneity and distribution of plasticizer molecules in plasticized PLA films were further evaluated by DMA, and the temperature dependence of tan δ for plasticized PLA films measured at 1 Hz is presented in Figure 8. The temperature dependences of the storage modulus and loss modulus are illustrated in Figure S17–S19. The peak value of tan δ, related to the glass transition, for neat PLA was 60 °C. The peak tan δ values were 45, 58, and 60 °C for the film plasticized by BTD-LeA, BTD-PLA-OH, and BTD-PLA-LeA, respectively, at 20 wt %. The observed peaks were wide and, in most cases, also exhibited some shoulders, suggesting an uneven distribution of plasticizers in the films, possibly due to the limited miscibility and/or the effect of crystalline regions. This broadness increased further when the plasticizer concentration was increased to 30 wt %. When the temperature was elevated to the melting temperature of BTD-LeA (*T*_m_ = 60 °C), another small peak in its tan δ curve was observed (Figure 8a). For the films plasticized by 20 wt % ISB-LeA and ISB-PLA-LeA, the peak tan δ values were 48 and 56 °C, respectively (Figure 8b). The tan δ curve of 20ISB-PLA-OH, without the functionalization of hydroxyl groups with LeA, exhibited a clear double peak at 50 and 59 °C as an indication of lower miscibility compared to that of ISB-PLA-LeA. Furthermore, at 30 wt % of the oligomeric plasticizers, wider and fluctuant tan δ curves with shoulder peaks were observed, indicating the probable existence of phase separation at high plasticizer concentrations (Figure 8a,b).

### Morphology of Fracture Surfaces

**Figure 9:**
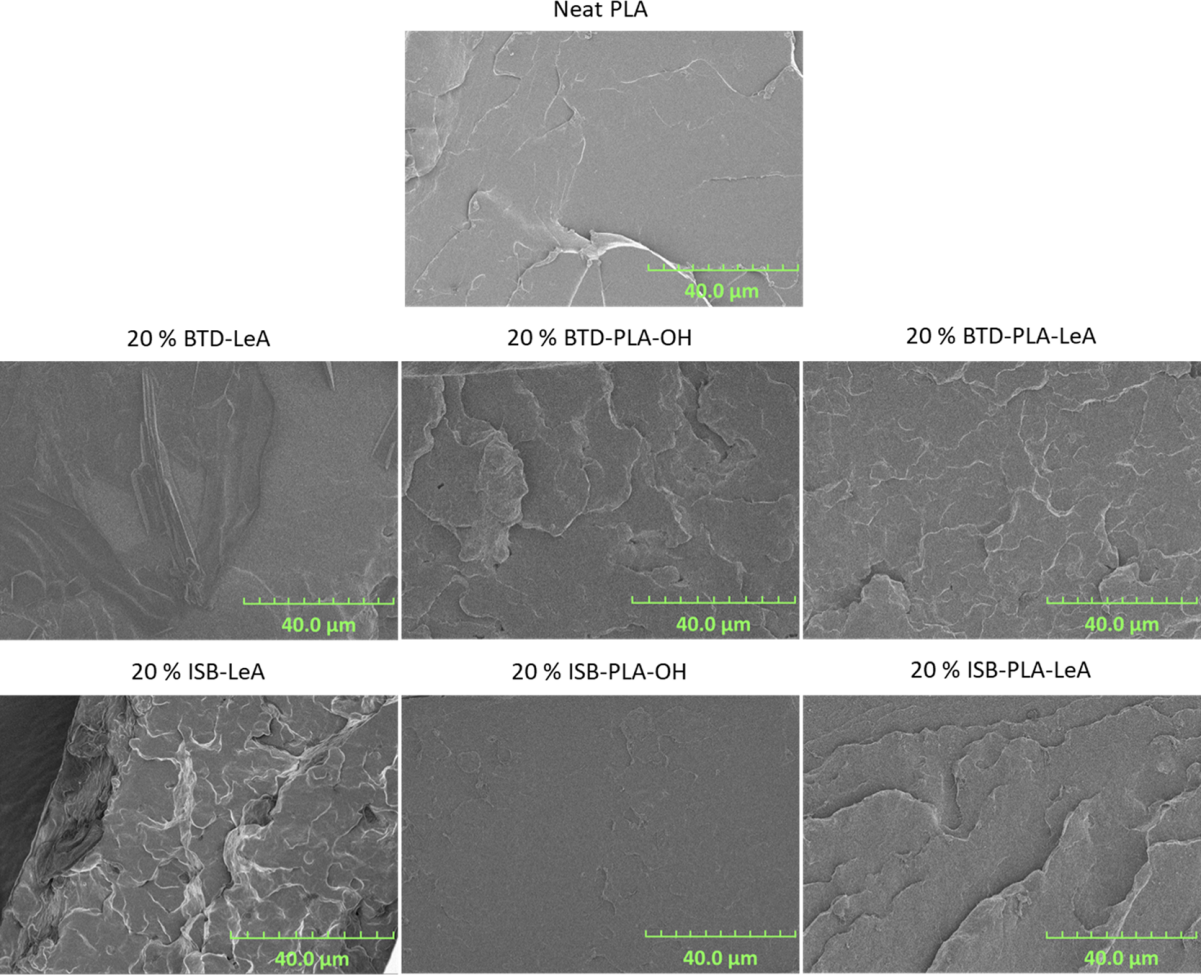
SEM images of neat PLA and PLA blends with 20 wt % of plasticizer candidates.

The morphology of the fracture surfaces of neat PLA and plasticized PLA films with different plasticizers were observed by scanning electron microscopy (SEM), and it is illustrated in Figures 9 and S20. The neat PLA demonstrated a smooth fracture surface due to its high brittleness. On the contrary, the PLA films plasticized by 20 wt % BTD-PLA-LeA, ISB-LeA, ISB-PLA-OH, or ISB-PLA-LeA exhibited more rough fracture surfaces, which is commonly observed for plasticized products.^6,44,45^ No voids or phase separation was observed in the films, suggesting that the four plasticizers were miscible with PLA. However, small voids were seen in 20BTD-PLA-OH, indicating the inferior miscibility of BTD-PLA-OH. Moreover, some clear and sharp crystals were captured in the SEM image of 20BTD-LeA, possibly due to BTD-LeA being prone to forming intergrown and twinned crystals.^46^ However, such crystals were not observed in 20 BTD-PLA-LeA, which indicates that the presence of oligolactide segments prevented the ability of the plasticizer to crystallize. When the plasticizer concentration increases and exceeds the limit of miscibility, phase separation will occur. As seen in Figure S20, at 30 wt % plasticizer concentration, phase separation, as indicated by the presence of voids, was found in PLA films plasticized by the four oligomeric plasticizers. Among them, the least voids were found in 30ISB-PLA-OH, implying the highest miscibility among the four oligomeric plasticizers. These findings support the indications from the DMA analysis.

### Hydrolytic Aging Study of the Plasticized PLA

The effect of the plasticizer and aging time on the degradation and migration patterns of the plasticized PLA blends was assessed by an accelerated hydrolytic aging test above the *T*_*g*_ of the materials, where the migrated plasticizers and the hydrolytic degradation products of PLA were fingerprinted by ESI-MS after predetermined aging periods. The mass loss and changes in molar mass were also monitored to supplement the migration pattern of the plasticized PLA. Neat PLA was used as a comparison in the test, and the overall results are summarized in Tables S2 and S3.

#### Identification of Migrated Plasticizers and PLA Oligomers from Plasticized PLA Films

**Figure 10:**
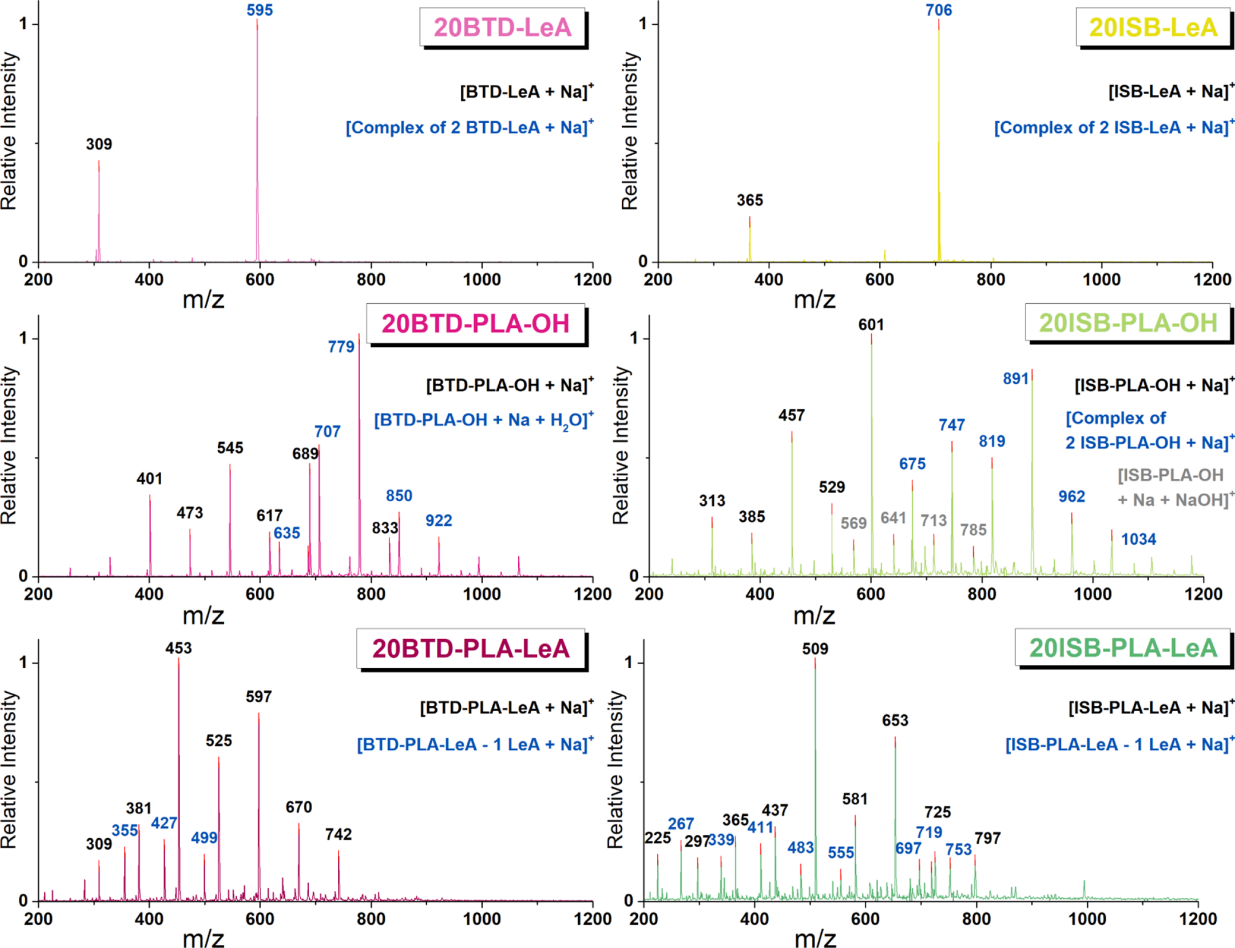
Migration products fingerprinted by ESI-MS of plasticized PLA films aged after 10 days.

All the plasticizers migrated to some extent after just 1 day of aging at 60 °C, similar to what has been observed previously.^22,47,48^ No PLA oligomers from the hydrolytic degradation of PLA were observed throughout the set time frame. The migrated compounds included the intact plasticizer molecules, a complex of plasticizer molecules, and hydrolyzed plasticizer molecules, as shown in ESI-MS spectra, as seen in Figure 10. All the products were detected as sodium adducts that sometimes were hydrated (e.g., BTD-PLA-OH and ISB-PLA-OH).

#### Mass Loss- and Aging-Induced Changes in Molar Mass

**Figure 11:**
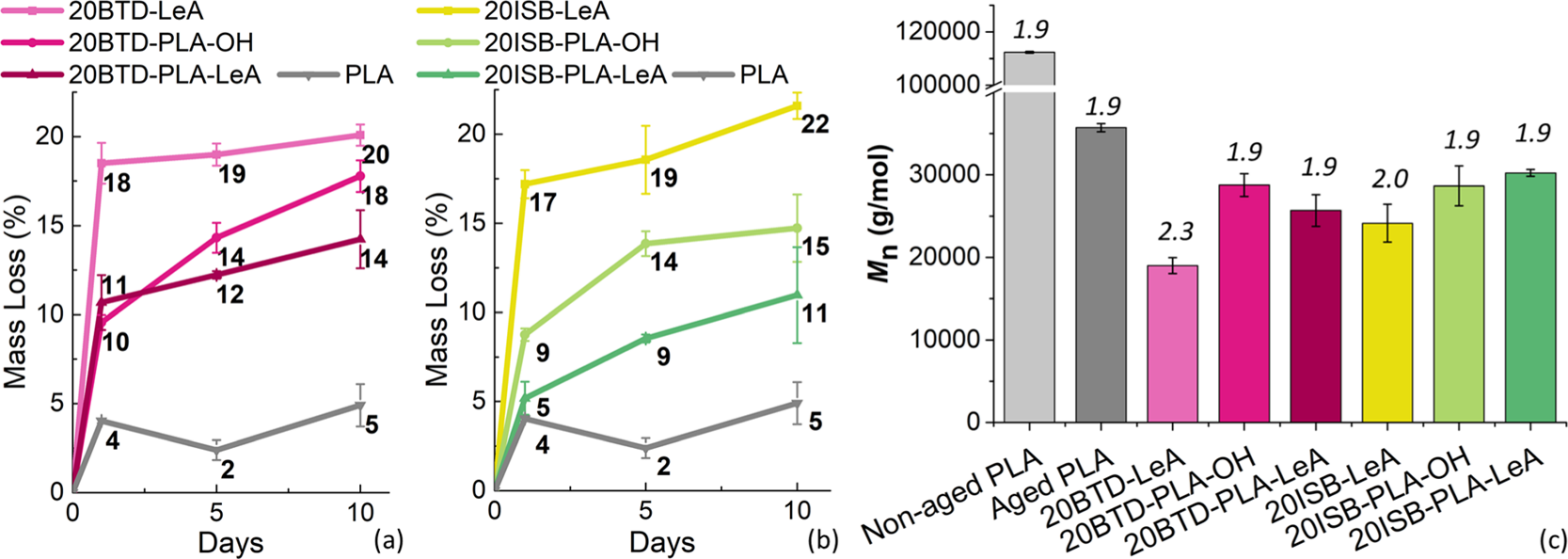
Mass loss as a function of aging time of neat PLA and PLA films containing BTD-based plasticizers (a), neat PLA and PLA films containing ISB-based plasticizers (b), and *M*_n_ and *D̵* (italic number) of neat PLA and plasticized PLA films aged after 10 days (c).

A fast migration of plasticizers occurred between days 0 and 1 of aging at the elevated temperature of 60 °C, and the migration continued during the remaining 9 days of aging but at a much slower rate, Figure 11a,b. The PLA films plasticized by monomeric plasticizers BTD-LeA and ISB-LeA exhibited higher rates of mass loss as compared to the four oligomeric plasticizers due to the lower molar mass, higher mobility, and also likely higher water solubility. The fast migration of plasticizers at the accelerated aging temperature above the *T*_*g*_ created empty spaces in the bulk of the plasticized PLA films that could be later filled by water molecules, which further accelerated the hydrolytic degradation of the PLA. This was proved by the fact that all the plasticized PLA films had significantly lower *M*_n_ than the aged neat PLA. A larger reduction in *M*_n_ was determined for 20BTD-LeA and 20ISB-LeA after 10 days of aging at 60 °C, as illustrated in Figure 11c and Table S3, compared to the blends with oligomeric plasticizers. Although the *M*_n_ of the four oligomeric plasticizers was in the same range, the variations in plasticizer structures influenced the hydrolytic aging pattern of the plasticized films. With the insertion of oligolactide segments and the functionalization of the hydroxyl groups with LeA, the plasticizer BTD-PLA-LeA and ISB-PLA-LeA demonstrated a dramatically retarded migration rate and the *M*_n_ of PLA was preserved to a higher degree during hydrolytic degradation, compared to the PLA films plasticized by BTD-PLA-OH and ISB-PLA-OH. Moreover, the alcohol core in the plasticizers likely affected the migration resistance of plasticizers. The PLA films plasticized by ISB-based plasticizers generally demonstrated slower rates of mass loss and equal or higher *M*_n_ after aging. This is explained by the cyclic and rigid ISB cores with larger sizes and more steric hindrance leading to decreased mobility as compared to flexible and linear BTD cores. Such a phenomenon was previously demonstrated by ketalized ED and ketalized GT plasticizers.^9^

## Conclusions

A series of monomeric and oligomeric plasticizers for PLA, with or without the inserted oligolactide segments, was successfully designed and synthesized by utilizing potentially biobased 1,4-butanediol and isosorbide cores and the green platform chemical levulinic acid. The structures of the designed plasticizes were confirmed by ^1^H NMR and ESI-MS. All the synthesized plasticizers decreased the *T*_g_ of PLA and the inserted oligolactide segments improved the migration resistance. At the same time, the inserted oligolactide segments slightly decreased the degree of plasticization, which is a generally observed phenomenon when the molar mass of the plasticizer increases. The flexible 1,4-butanediol core and end-group functionalization with LeA were defined as two beneficial factors for efficient plasticization of PLA, while a rigid isosorbide core and hydroxyl end groups contributed to higher values of the Young’s modulus and stress at break. Moreover, the end-group functionalization with LeA and the presence of oligolactide segments both increased the thermal stabilities of plasticizers and when combined in plasticizer design, superior migration resistance was obtained in a hydrolytic aging test. In summary, the incorporation of oligolactide segments, end-group functionalization with LeA, and core selection in the plasticizer design were demonstrated as a set of routes to tailor miscibility and plasticizer performances.

## Supporting Information Available

NMR spectra of the five synthesized plasticizers, thermal properties of neat PLA and plasticized PLA films (including theoretical *T*_g_ values calculated by the Fox equation), tensile curves and DMA curves of neat PLA and plasticized PLA films, SEM images of fracture surface of PLA films plasticized by 30 wt % oligomeric plasticizers, ESI-MS results, and size exclusion chromatography results (PDF)
