# Supplementary material for: Tailoring Oligomeric Plasticizers for Polylactide through Structural Control
Source: ACS Omega. 2022 Apr 12;7(16):14305–16. doi: 10.1021/acsomega.2c01160 (PMC9089748; doi:10.1021/acsomega.2c01160)
Supplement: Supplementary file 1 — ao2c01160_si_001.pdf [file ao2c01160_si_001.pdf]

Supporting Information

Tailoring Oligomeric Plasticizers for Polylactide through Structural Control

Wenxiang Xuan, Karin Odelius and Minna Hakkarainen*

Kungliga Tekniska Högskolan, KTH, Department of Fibre and Polymer Technology, Teknikringen 58, SE 10044 Stockholm, Sweden

*Corresponding author: Minna Hakkarainen, E-mail address: minna@kth.se

Number of pages: 13

Number of figures: 20

Number of tables: 3

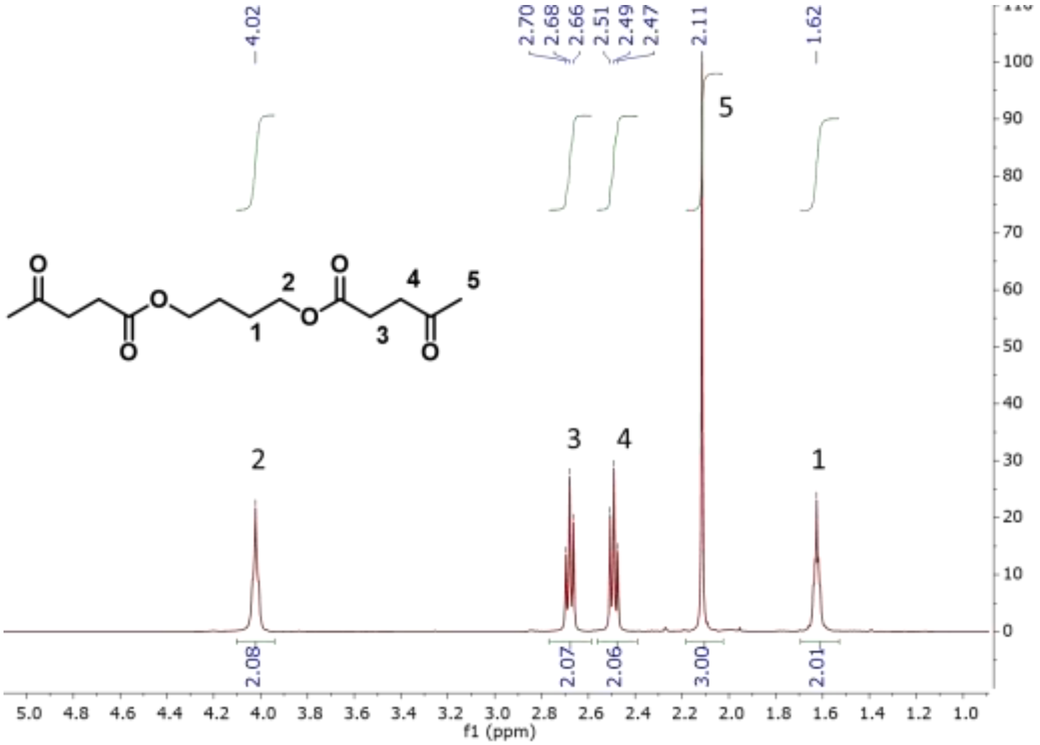

Figure S1. ^{1}H NMR spectrum of BTD-LeA

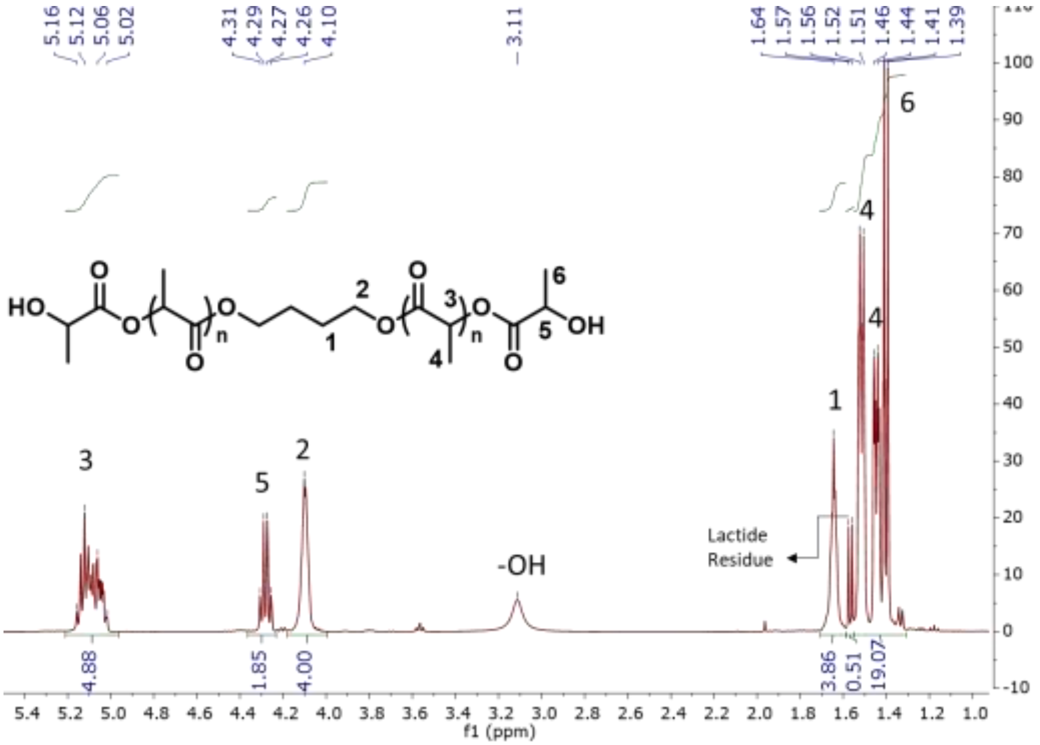

Figure S2. ^{1}H NMR spectrum of BTD-PLA-OH

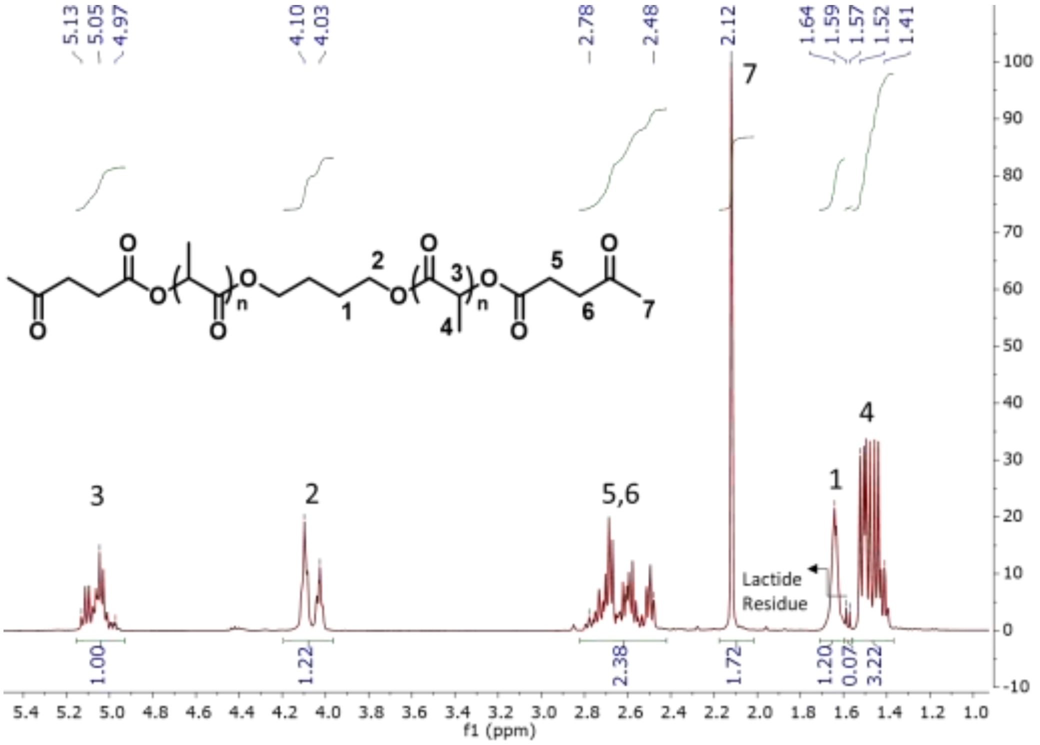

Figure S3. ^{1}H NMR spectrum of BTD-PLA-LeA

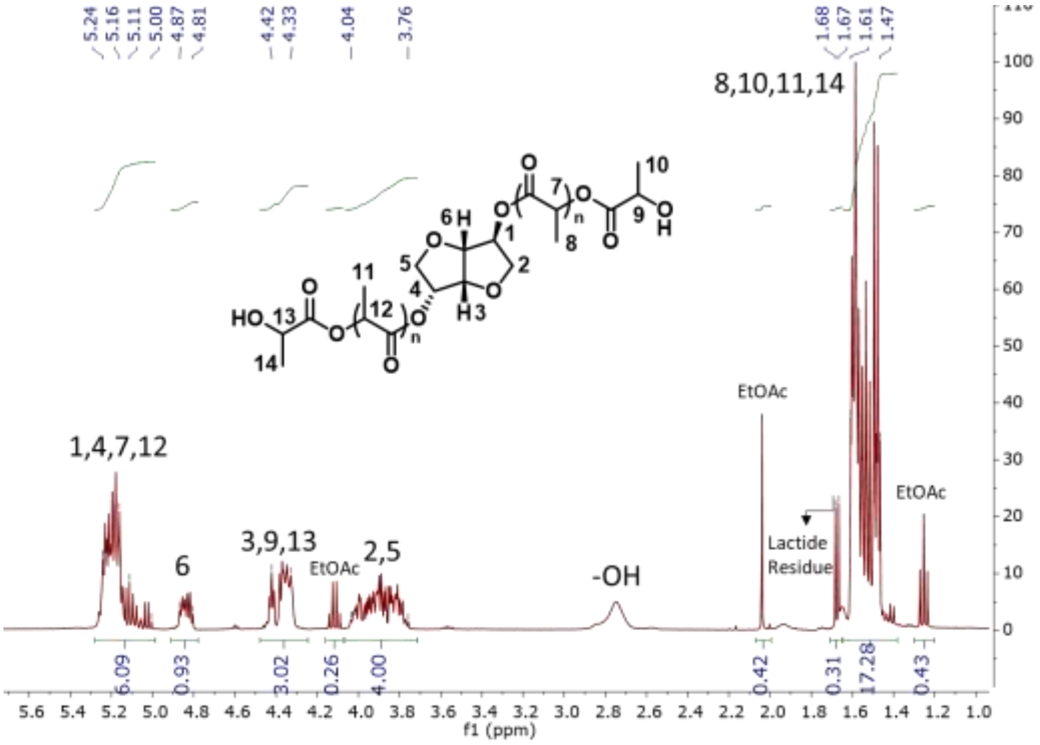

Figure S4. ^{1}H NMR spectrum of ISB-PLA-OH

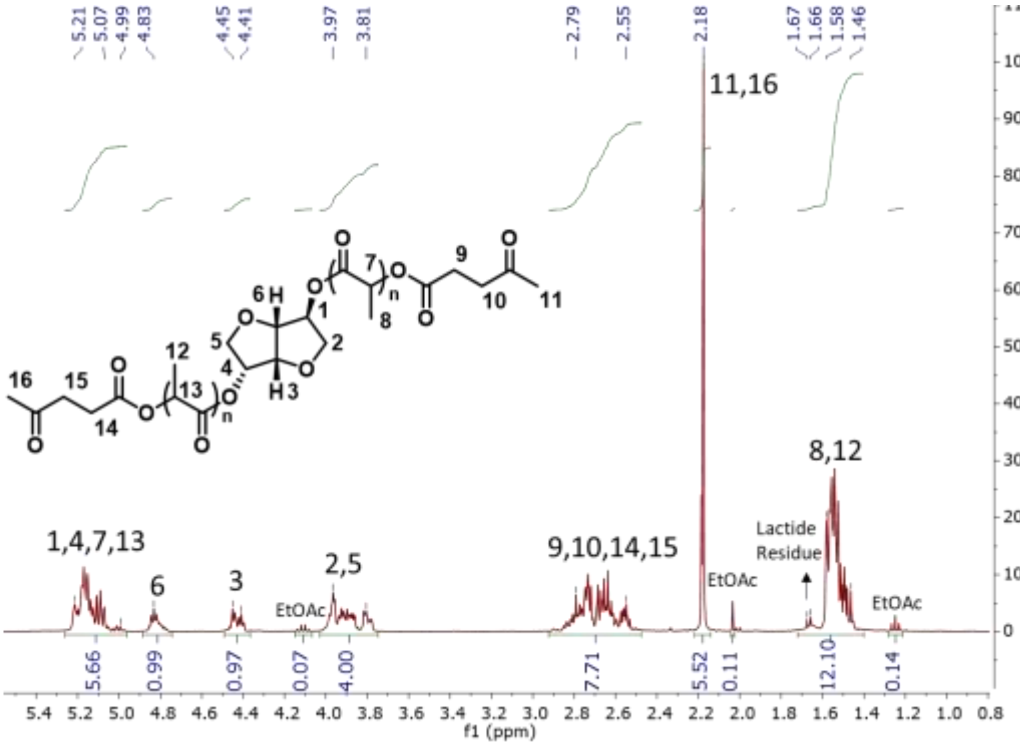

Figure S5. ^{1}H NMR spectrum of ISB-PLA-LeA

Table S1. Thermal properties of neat PLA and plasticized PLA films containing 20 wt% and 30 wt% plasticizers (theoretical T_g values were obtained by applying Fox equation ($1/T_{g,mix} \approx \omega_{PLA}/T_{g,PLA} + \omega_{plasticizer}/T_{g,plasticizer}$).

	T_g^a (°C)	T_{cc}^a (°C)	T_m^a (°C)	χ_c^a (%)	χ_c^b (%)	Theor. T_g^c (°C)
Neat PLA	59 ± 0.1	N/A	N/A	N/A	31 ± 1.3	N/A
20BTD	16 ± 0.8	99 ± 1.6	140 ± 0.1	N/A	22 ± 0.7	N/A
20BTD-PLA-OH	35 ± 0.6	N/A	142 ± 0.1	1 ± 0.2	25 ± 0.5	42
20BTD-PLA-LeA	28 ± 0.6	N/A	140 ± 0.2	2 ± 0.1	24 ± 1.7	35
20ISB-LeA	35 ± 0.1	N/A	N/A	N/A	23 ± 1.0	38
20ISB-PLA-OH	45 ± 0.4	N/A	N/A	N/A	26 ± 0.6	48
20ISB-PLA-LeA	42 ± 0.5	N/A	N/A	N/A	24 ± 1.0	46
30BTD-PLA-OH	24 ± 1.7	110 ± 2.0	136 ± 0.2	0.5 ± 0.4	21 ± 1.7	32
30BTD-PLA-LeA	18 ± 0.2	108 ± 0.2	136 ± 0.0	0.1 ± 0.1	24 ± 0.4	24
30ISB-PLA-OH	39 ± 0.3	N/A	N/A	N/A	21 ± 0.6	42
30ISB-PLA-LeA	33 ± 0.5	N/A	N/A	N/A	22 ± 0.8	40

a:data from the second heating scan; b:data from the first heating scan; c: T_g from DSC was – 17 ± 0.3 °C for BTD-PLA-OH, – 36 ± 0.4 °C for BTD-PLA-LeA, – 26 ± 0.5 °C for ISB-LeA, 9 ± 0.4 °C for ISB-PLA-OH and 2 ± 0.4 °C for ISB-PLA-LeA.

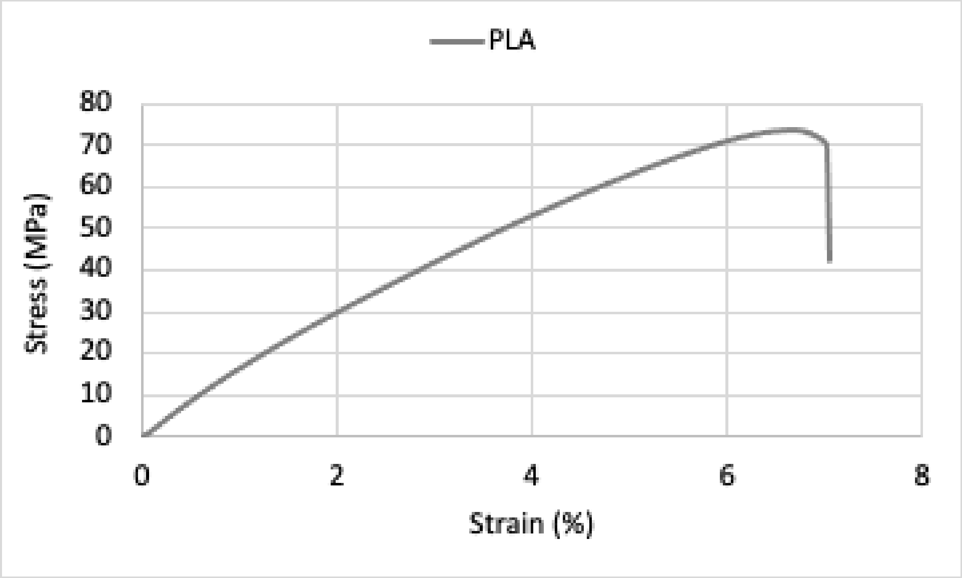

Figure S6. Tensile curve of neat PLA film.

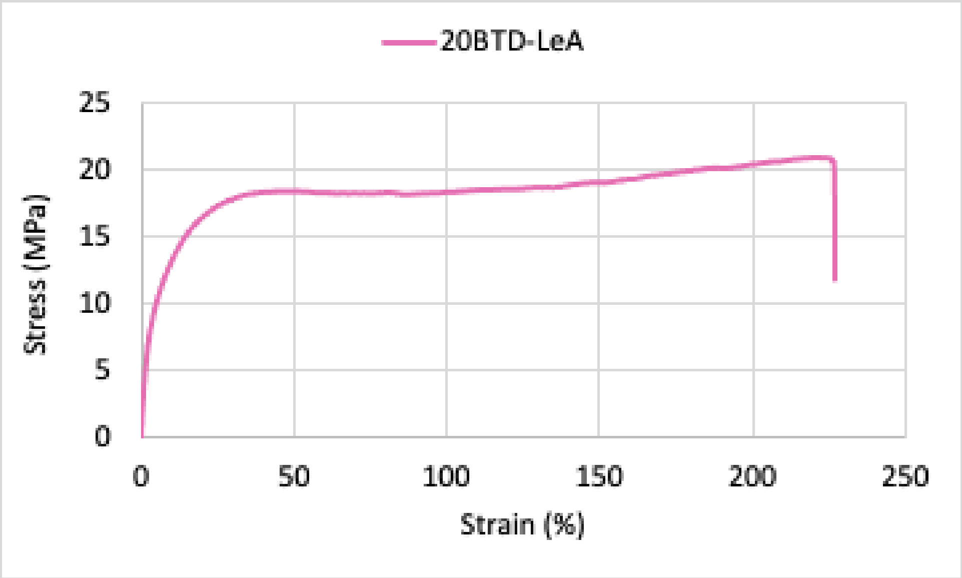

Figure S7. Tensile curve of film 20BTD-LeA.

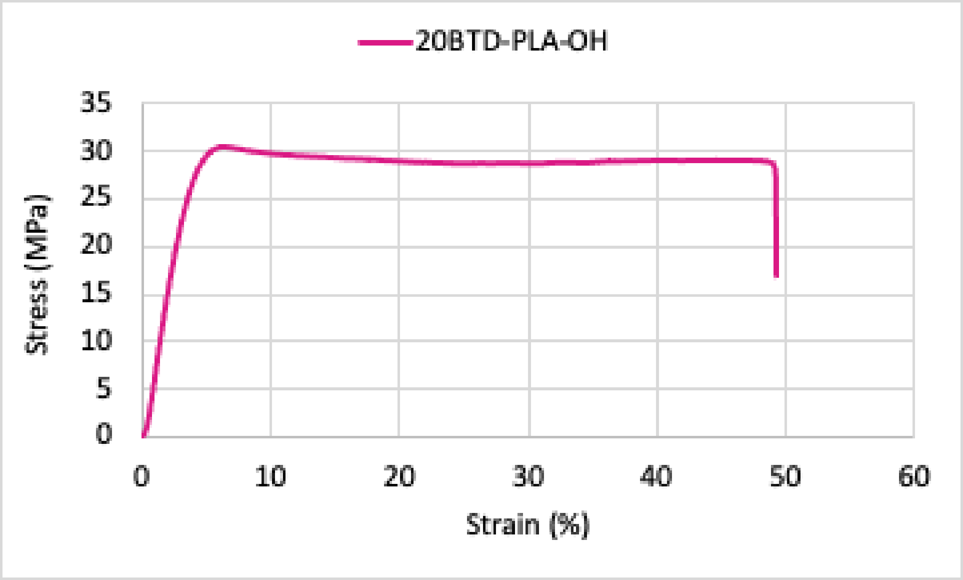

Figure S8. Tensile curve of film 20BTD-LeA-OH.

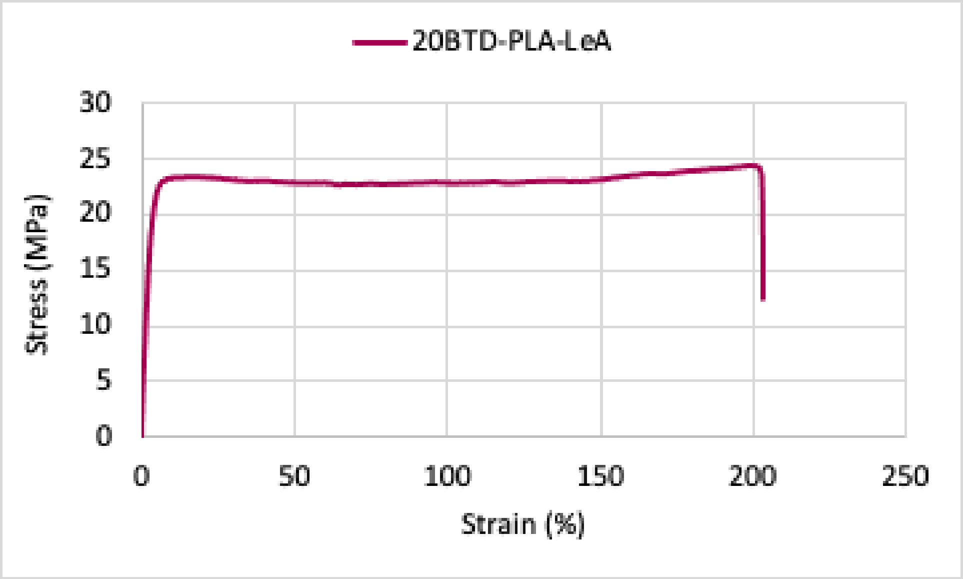

Figure S9. Tensile curve of film 20BTD-PLA-LeA.

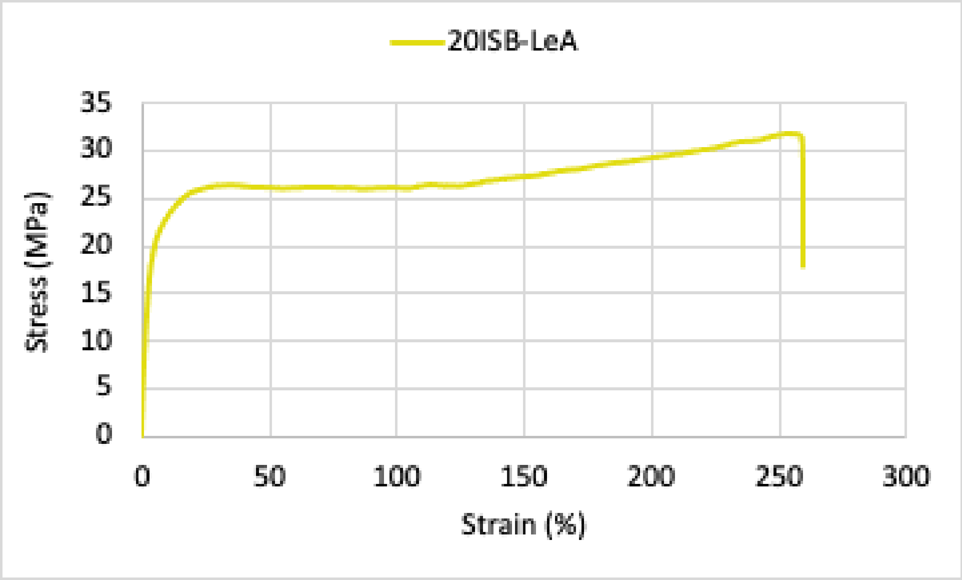

Figure S10. Tensile curve of film 20ISB-LeA.

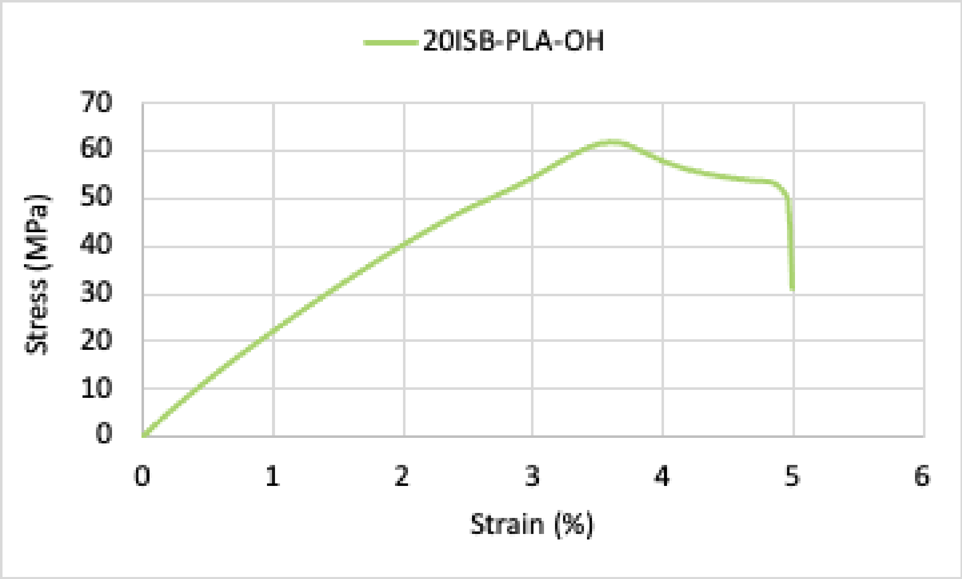

Figure S11. Tensile curve of film 20ISB-PLA-OH.

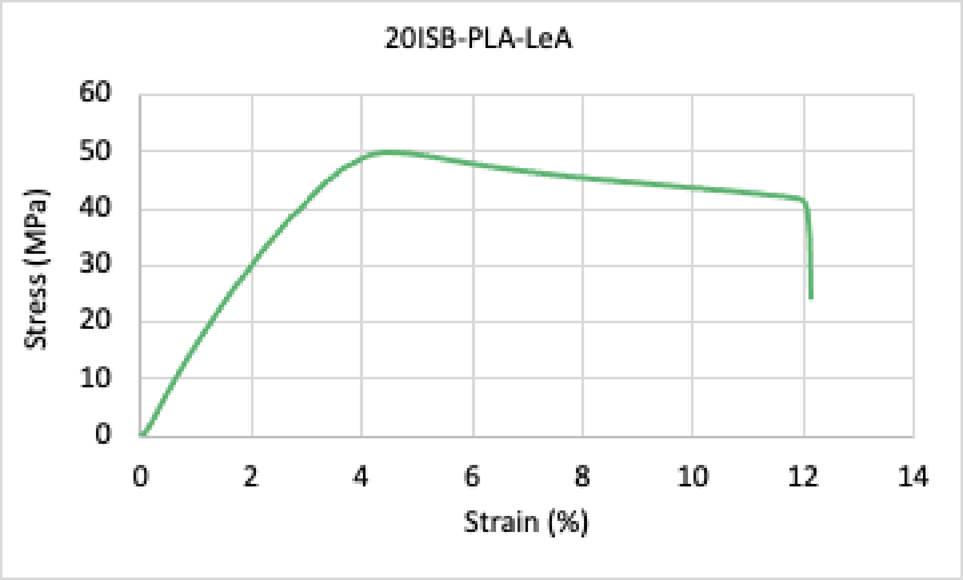

Figure S12. Tensile curve of film 20ISB-PLA-LeA.

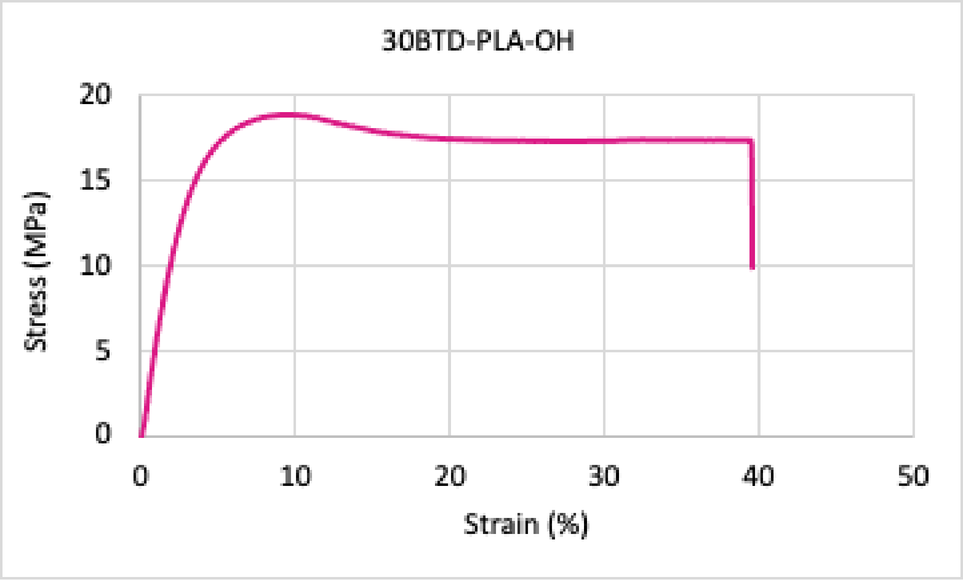

Figure S13. Tensile curve of film 30BTD-PLA-OH.

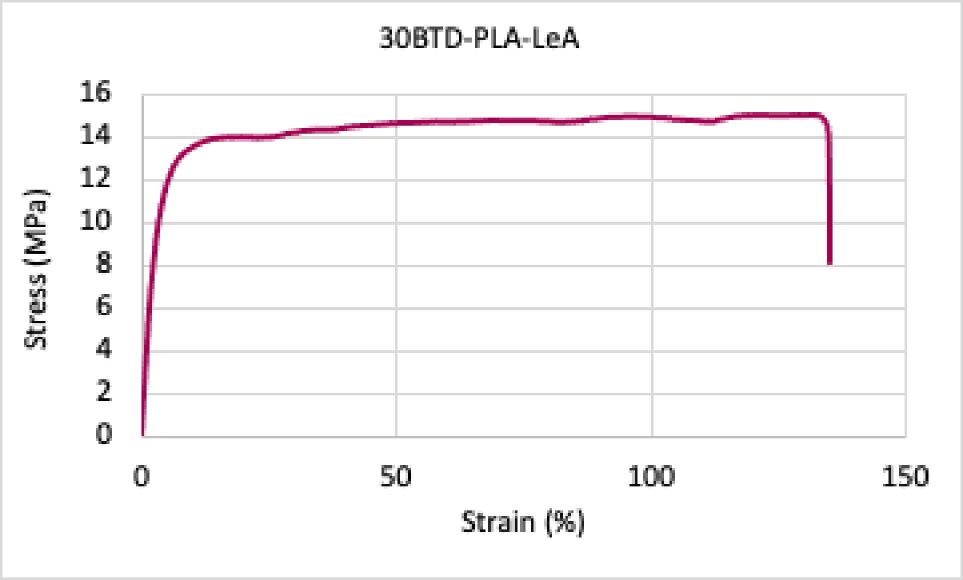

Figure S14. Tensile curve of film 30BTD-PLA-LeA.

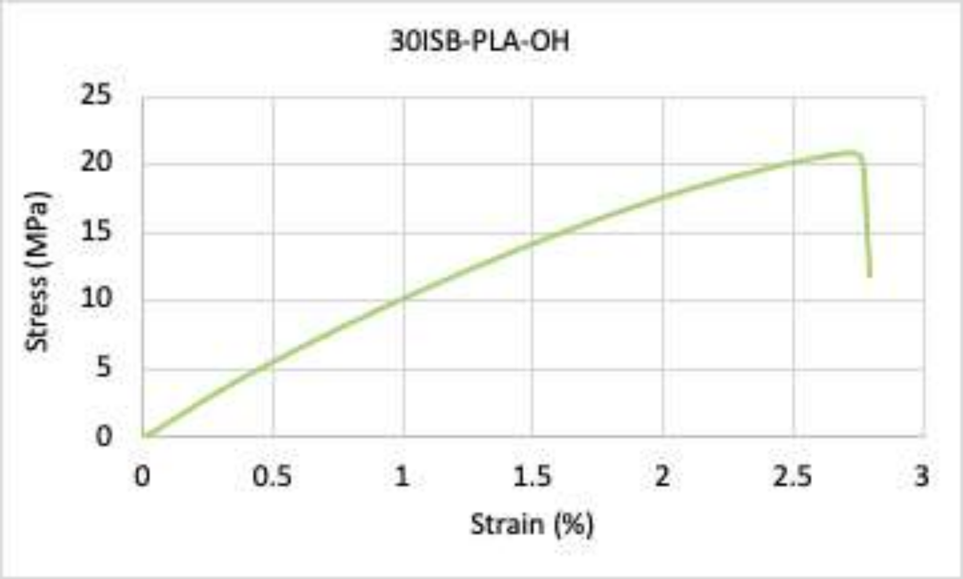

Figure S15. Tensile curve of film 30ISB-PLA-OH.

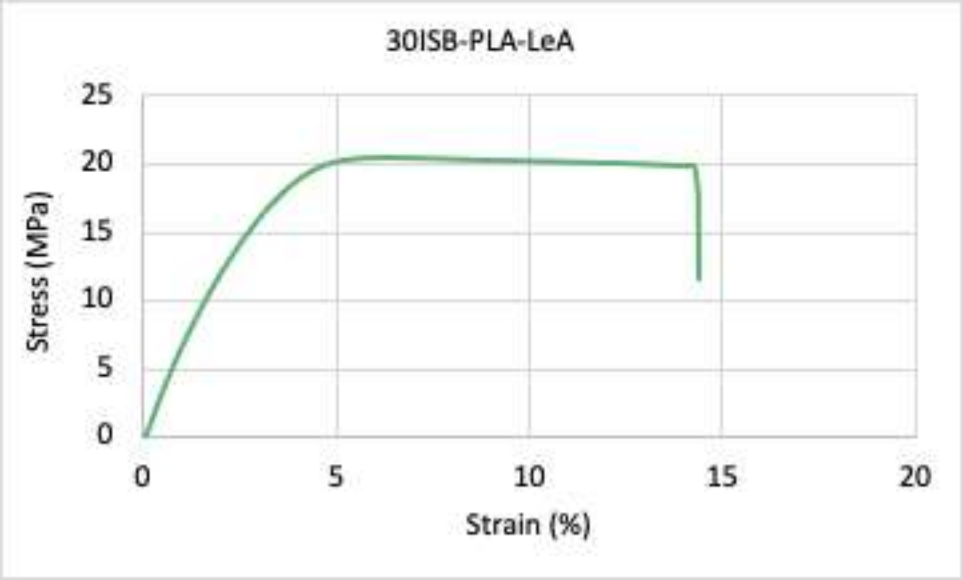

Figure S16. Tensile curve of film 30ISB-PLA-LeA.

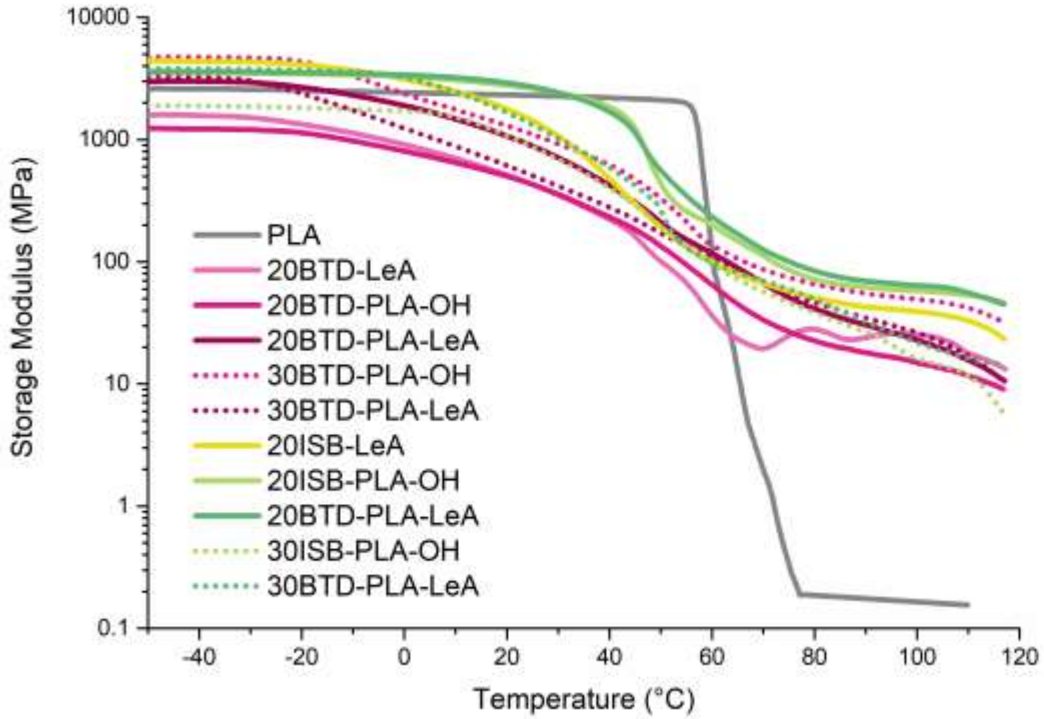

Figure S17. Temperature dependence of storage modulus for PLA films at 1 Hz.

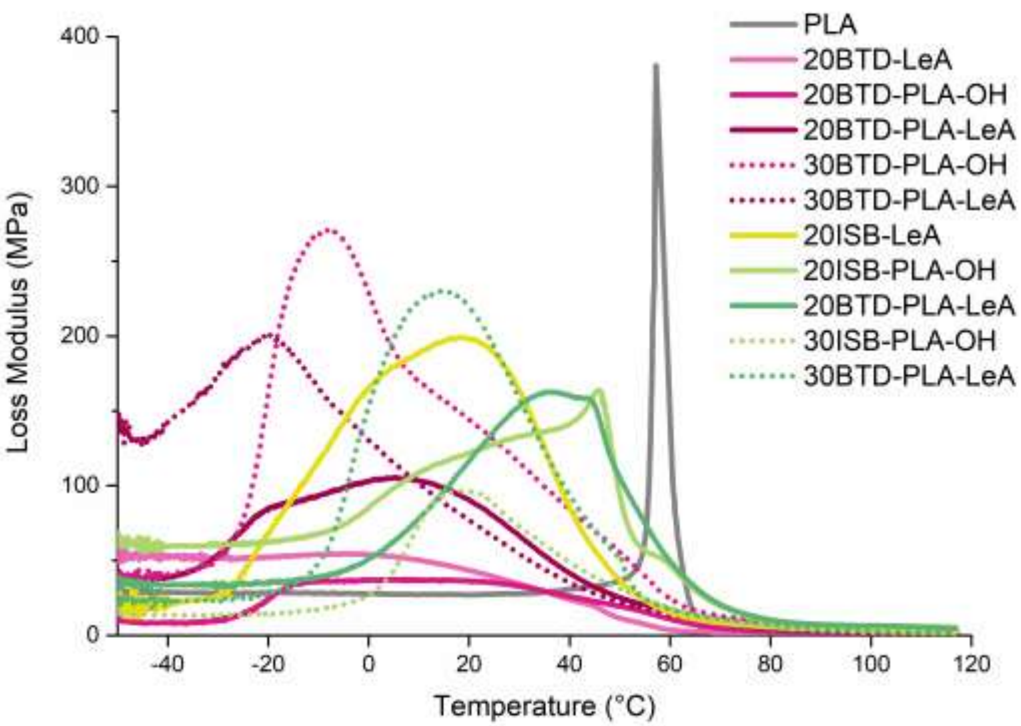

Figure S18. Temperature dependence of loss modulus for PLA films at 1 Hz.

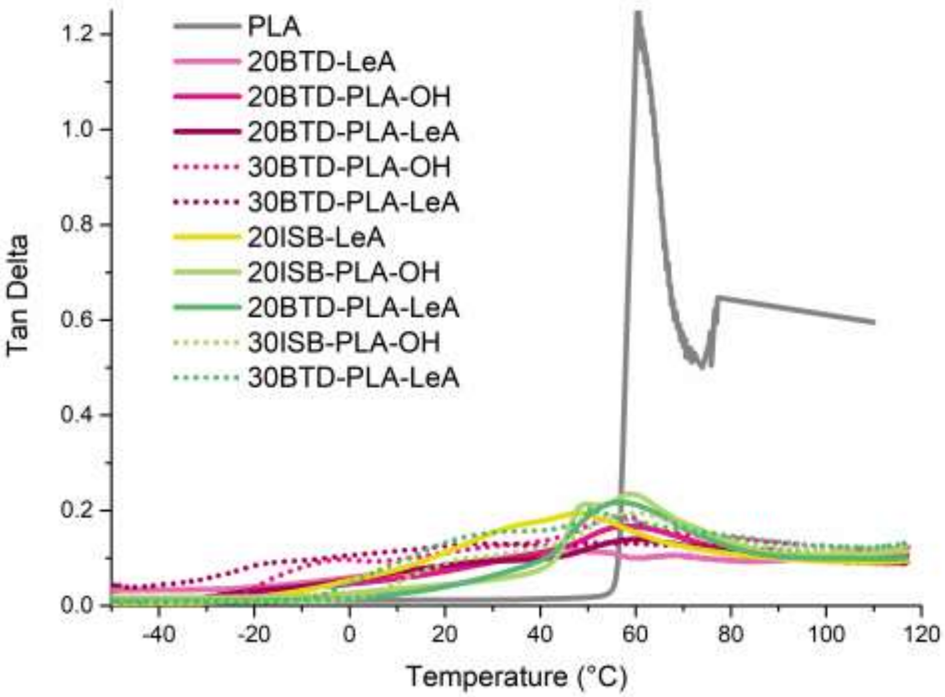

Figure S19. Temperature dependence of tan δ for PLA films at 1 Hz.

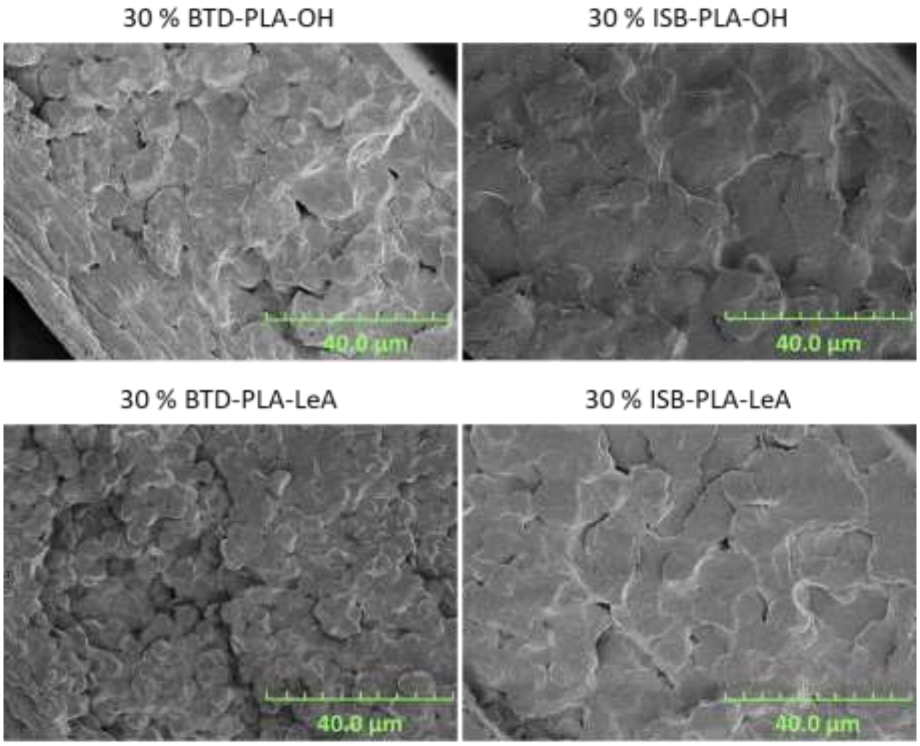

Figure S20. SEM images of PLA films plasticized by 30 wt% oligomeric plasticizers.

Table S2. Summary of ESI-MS fingerprinting result of neat PLA and plasticized PLA films after 10 days at 60 °C.

	1 Day		5 Days		10 Days	
Sample	Plasticizer or Oligomer	PLA	Plasticizer or Oligomer	PLA	Plasticizer or Oligomer	PLA
PLA	-	-	-	-	-	-
20BTD	+	-	+	-	+	-
20BTD-PLA-OH	+	-	+	-	+	-
20BTD-PLA-LeA	+	-	+	-	+	-
20ISB-LeA	+	-	+	-	+	-
20ISB-PLA-OH	+	-	+	-	+	-
20ISB-PLA-LeA	+	-	+	-	+	-

Note: +, detected in ESI MS; -, not detected in ESI MS.

Table S3. SEC of neat PLA and plasticized PLA films aged after 10 days at 60 °C.

Sample	M_n (g/mol)	Đ
PLA (before hydrolysis)	112,000 ± 300	1.9
PLA	36,000 ± 500	1.9
20ISB-LeA	24,000 ± 2,000	2.0
20ISB-PLA-OH	29,000 ± 2,000	1.9
20ISB-PLA-LeA	30,000 ± 400	1.9
20BTD-LeA	19,000 ± 1,000	2.3
20BTD-PLA-OH	29,000 ± 1,000	1.9
20BTD-PLA-LeA	26,000 ± 2,000	1.9
